# Clinical Efficacy and Safety of Bevacizumab Monotherapy in Patients with Metastatic Melanoma: Predictive Importance of Induced Early Hypertension

**DOI:** 10.1371/journal.pone.0038364

**Published:** 2012-06-15

**Authors:** Cornelia Schuster, Hans P. Eikesdal, Hanne Puntervoll, Jürgen Geisler, Stephanie Geisler, Daniel Heinrich, Anders Molven, Per E. Lønning, Lars A. Akslen, Oddbjørn Straume

**Affiliations:** 1 Department of Oncology, Haukeland University Hospital, Bergen, Norway; 2 Section for Pathology, The Gade Institute, University of Bergen, Bergen, Norway; 3 Department of Pathology, Haukeland University Hospital, Bergen, Norway; 4 Division of Clinical Medicine and Laboratory Sciences, Institute of Clinical Medicine, University of Oslo, Oslo, Norway; 5 Department of Oncology, Akershus University Hospital, Lørenskog, Norway; The Moffitt Cancer Center & Research Institute, United States of America

## Abstract

**Background:**

VEGF driven angiogenesis plays a key role in cancer progression. We determined the clinical efficacy of bevacizumab monotherapy in patients with metastatic melanoma.

**Methods and Findings:**

Thirty-five patients with metastatic melanoma in progression were enrolled in this phase II, single arm clinical trial. Each patient received bevacizumab monotherapy 10 mg/kg q14 d until intolerable toxicity or disease progression occurred. Clinical efficacy was evaluated as objective response, disease control (DC), and survival. We observed one complete (3%) and 5 partial (14%) responses. In addition, 5 patients experienced stable disease >6 months (14%) while 24 patients had progressive disease (PD, 69%), corresponding to a total DC at 6 months in 11 out of 35 patients (31%). Median progression free survival (PFS) was 2.14 months and median overall survival (OS) was 9 months (1.12–49). Seven of the 11 patients experiencing DC developed early hypertension (<2 months) compared to 3/24 of patients with PD (*P* = 0.001), and hypertension was associated with PFS (*P* = 0.005) and OS (*P* = 0.013).

**Conclusion:**

Bevacizumab monotherapy demonstrated promising clinical efficacy in patients with metastatic melanoma with disease control in 31% of the patients. Induced early hypertension was a marker for clinical efficacy of bevacizumab.

**Trial Registration:**

ClinicalTrials.gov NCT00139360.

## Introduction

Metastatic melanoma is a non-curable condition with limited therapeutic options. Until recently, high dose interleukin-2 and dacarbazine were the only regimens in routine use, with response rates observed in about 10% of unselected patients[1]–[3]. While the human monoclonal anti CTLA-4 antibody ipilimumab was recently shown to cause a survival benefit in stage IV melanoma [4], the drug was found active in a fraction of patients only. Improved survival was also reported for treatment of metastatic melanoma patients carrying a specific *BRAF* mutation (∼40% of all melanoma patients) using the highly selective V600E kinase inhibitor vemurafenib [5]. Thus, while selected patients may benefit from novel treatment options, effective treatment is still not available for a high proportion of melanoma patients. In addition, patients benefitting from conventional (interleukin-2 or dacarbazine) as well as novel (ipilimumab and vemurafenib) therapeutic strategies develop acquired therapy resistance over time, underlining the need for alternative treatment options.

Melanoma progression and metastasis is dependent on angiogenesis [6] and the vascular endothelial growth factor (VEGF) system seems to be particularly important [7], [8]. The humanized monoclonal antibody bevacizumab is a highly specific inhibitor of VEGF-A. Bevacizumab significantly prolonged overall survival when given in combination with chemotherapy in colorectal cancer [9] and in non-small cell lung cancer [10]. In addition, responses have been reported in clinical trials evaluating bevacizumab in combination with interferon alpha 2B [11], interferon alpha 2A [12] or chemotherapy[13]–[15] in patients with metastatic melanoma. Administered as monotherapy, bevacizumab prolonged time to progression given in patients suffering from metastatic kidney cancer [16].

To the best of our knowledge, no clinical trials have been published specifically testing the clinical efficacy of bevacizumab monotherapy in metastatic melanoma. Here, we report the results from a phase II trial evaluating clinical efficacy of bevacizumab monotherapy in patients with metastatic melanomas.

## Methods

### Ethics

The study was conducted in accordance with the ethical principles of the Declaration of Helsinki and the International Conference on Harmonization of Good Clinical Practice. The protocol was approved by the Regional Ethics Committee and the Norwegian Medicines Agency. All participating patients provided signed informed consent before enrolment.

### Patients

Between April 2005 and August 2009, 52 patients were screened. Eligibility criteria included histologically confirmed unresectable metastatic melanoma in progression; age >18 years; WHO performance status 0–2; clinically and/or radiographically measurable disease according to RECIST; >4 weeks since adjuvant interferon; no prior interferon or interleukin for metastatic disease; recovered from prior chemotherapy; no major surgery within 28 days; no known brain metastases; absolute neutrophils >1.0×10^9^/L; platelets >100×10^9^/L; bilirubin, creatinine, INR <1.5×upper normal limit; no symptomatic congestive heart failure, angina pectoris, cardiac arrhythmia, history of thrombosis, uncontrolled hypertension, full dose coumarin-derived anticoagulants or NSAIDS.

### Study Design

This was a phase II, open-label, single-arm, single institution clinical trial (ClinicalTrials.gov Identifier: NCT00139360), performed at the Haukeland University Hospital, Bergen Norway. The full protocol is available online as supporting information (Protocol S1). The primary objective was to determine clinical efficacy, as measured by objective response (OR) and disease control (DC) defined as stable disease (with or without an objective tumor shrinkage) after 6 months on therapy. Secondary objectives were to estimate time to progression (TTP), progression free survival (PFS) and overall survival (OS). Finally, we aimed at exploring potential relations between side effects, including acquired hypertension as well as *BRAF/NRAS* mutation status as potential predictive factors to clinical response.

Initially, patients were included after confirmed progression on standard first line treatment with dacarbazine (level A, n = 15). Only after objective response was observed on bevacizumab monotherapy, all new patients were subsequently enrolled for first line treatment with bevacizumab (Level B, 20 patients) (Flow diagram S1).

Each treatment cycle consisted of bevacizumab 10 mg/kg IV on day 1 in a 2-weekly schedule. Thus, the chosen dose was higher than the doses used in bevacizumab therapies for normalization of tumor vasculature (5 mg/kg q14d) [17], [18] and in line with the dosing of bevacizumab monotherapy used in advanced renal cancer where a survival benefit was indicated (10 mg/kg q14d) [16]. Drug toxicity was assessed after each cycle, while the response rate was evaluated after every 4 cycles. Patients with disease progression or unmanageable toxicity were discontinued and offered further melanoma treatment at the clinician’s discretion. Standard clinical parameters (routine biochemistry, urine analysis, blood pressure, WHO performance status) as well as the mutational status for *BRAF* and *NRAS* were assessed for subsequent correlation with clinical outcome.

### Response Assessment and Toxicity

The primary endpoint was objective response (OR) defined as complete response (CR) or partial response (PR) according to RECIST [19] as well as disease control (DC) defined as CR + PR and including stable disease (SD) for more than 6 months. Disease stabilization is considered beneficial to patients experiencing melanoma progression at the time of inclusion and DC is frequently included as an additional statistical endpoint in trials investigating new antiangiogenic drugs in which therapeutic activity and clinical benefit are present, even in the absence of radiological tumor shrinkage[20]–[22]. Importantly, all patients were in clinical and/or radiological progression at the time of inclusion. OR and DC were calculated on the basis of investigator assessment. While confirmed response after 4 weeks was not a protocol requirement, all patients achieving an objective response had a subsequent confirmation at the next routine visit every 8 weeks. Patients with clinical disease progression or death due to melanoma before first radiological progression were recorded as progressive disease (PD), and best overall response (BOR) was not available in these patients. TTP was defined as the time from enrolment to disease progression or death due to melanoma.

Adverse events were graded according to the National Cancer Institute Common Terminology Criteria for Adverse events, version 3.0 [23], and were recorded by each 2-week cycle.

### Tissue Sampling and DNA Analysis

To evaluate a possible relationship between the most frequent genetic alterations in melanoma and treatment outcome, a targeted mutational analysis was performed for *BRAF* and *NRAS*. Tumor tissue was manually dissected from 3 paraffin sections (10 µm) before extracting DNA with the E.Z.N.A Tissue DNA Kit (Omega Bio-Tek, Inc., Norcross, GA, USA). *BRAF* exon 11 and 15, as well as *NRAS* exon 1 and 2 were amplified by PCR, and screened for mutations by direct Sanger sequencing. Primers are described elsewhere[24]–[26]. The sequence reactions were performed using the Terminator Cycle Sequencing kit, BigDye version 1.1 (Applied Biosystems, Foster City, CA, USA ), and were analyzed on an ABI PRISM® 3100 Genetic Analyzer, applying Sequencing Analysis software, version 3.7 (both from Applied Biosystems).

### Statistical Methods

The optimal two-stage design for phase II clinical trials proposed by Simon [27] was used. The co-primary endpoint DC was used to determine sample size. It was assumed that the new regimen would have a DC rate of 30%. A DC rate of 10% or lower was considered not superior to standard first-line therapy (dacarbazine). With 10% type I error rate and 90% power a total number of 35 patients were entered in the trial.

Two sample t-test and Mann-Whitney U test were used to compare the distribution of continuous variables between two groups such as responders and non-responders. Kaplan-Meier estimates were constructed for time-to-event endpoints such as PFS and OS, and log rank-test was applied for testing differences. Due to the small sample size and the nature of the phase II study, the above analyses were considered exploratory and the results need to be confirmed in future large-scale studies.

## Results

### Patients

**Figure 1:**
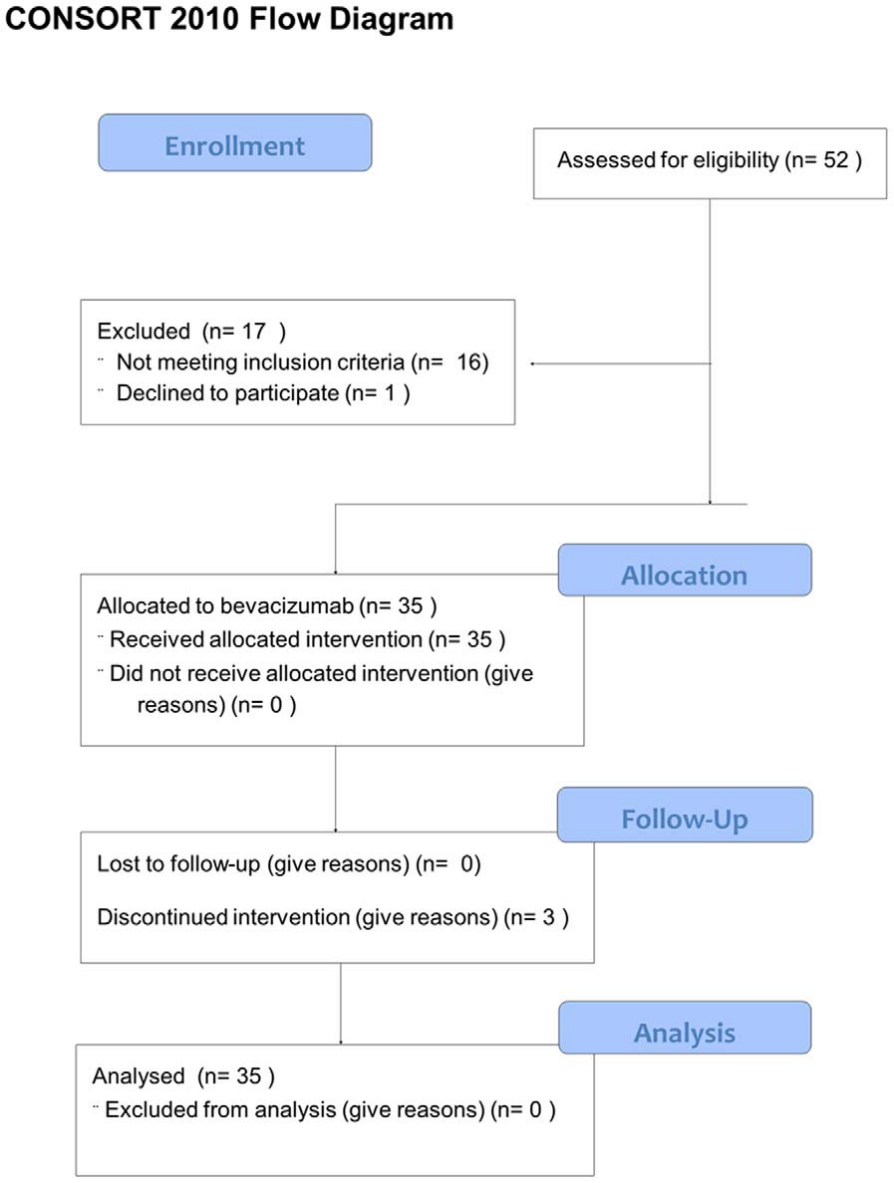
Study flow diagram. Between April 2005 and August 2009, 52 patients with metastatic melanoma were screened. Thirty-five of those patients were eligible according to inclusion criteria and received the study drug.

Between April 2005 and August 2009, 52 patients with metastatic or unresectable melanoma in progression were screened and 35 patients were enrolled in this trial. The seventeen screening failures were most frequently due to brain metastases, co morbidity, or withdrawal of informed consent (Fig 1). During recruitment at level A, 15 patients received bevacizumab as second/third line treatment (after DTIC failure) while additional 20 more patients were included during recruitment level B (first line therapy bevacizumab). Patient characteristics are listed in Table 1.

**Table 1:** Baseline Demographic and Clinical Characteristics of Patients.

Characteristics	Study cohort (*n* = 35)
Age, years	
Median	63
Range	26–77
Sex - No. (%)	
Male	19 (54)
Female	16 (46)
Stage - No. (%)	
M1a	1 (3)
M1b	6 (17)
M1c	28 (80)
LDH>ULN - No. (%)	
No	14 (40)
Yes	21 (60)
WHO performance status - No. (%)	
0	28 (80)
1	7 (20)
Previous systemic treatments - No. (%)	
0	20 (57)
1	14 (40)
2	1 (3)
Hypertension before treatment - No. (%)	
No	27 (77)
Yes	8 (23)
*BRAF* exon 15 mutation – No. (%)	
Wild type	20 (57)
V600E	13 (37)
V600K	1 (3)
V600D/V600E Double mutation	1 (3)
*NRAS* exon 2 mutation - No. (%)	
Wild type	24 (69)
Q61R	4 (11)
Q61L	2 (6)
Q61K	3 (9)
E62E	1 (3)
Not amplifiable	1 (3)

Abbreviations: LDH, lactate dehydrogenase; ULN, upper limit of normal; WHO, World Health Organization.

### Responses, PFS and Survival

**Figure 2:**
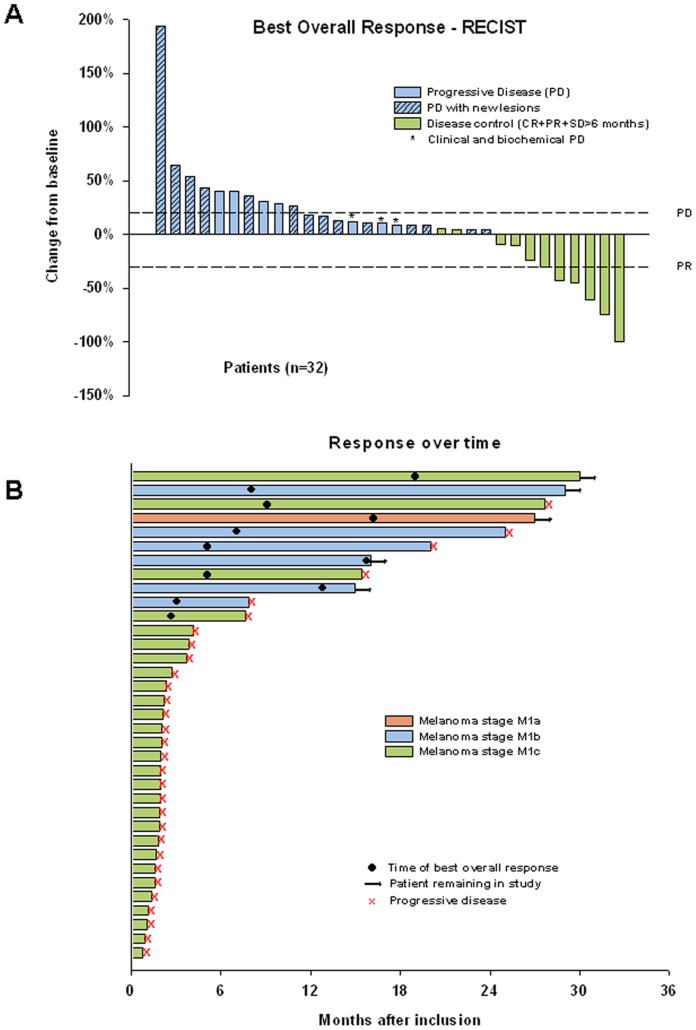
Patterns of response to treatment with bevacizumab monotherapy in metastatic malignant melanoma patients. Panel A shows the best overall response for 32 patients who had undergone at least one tumor assessment measured as the change from baseline in the sum of the largest diameters of each target lesion. Three patients progressed clinically and/or biochemically before first tumor assessment, and are not shown. Negative values indicate tumor shrinkage, and the dashed lines indicate the threshold for a partial response (PR) and progressive disease (PD), respectively. Panel B shows the duration and characteristics of the responses in each patient.

**Figure 3:**
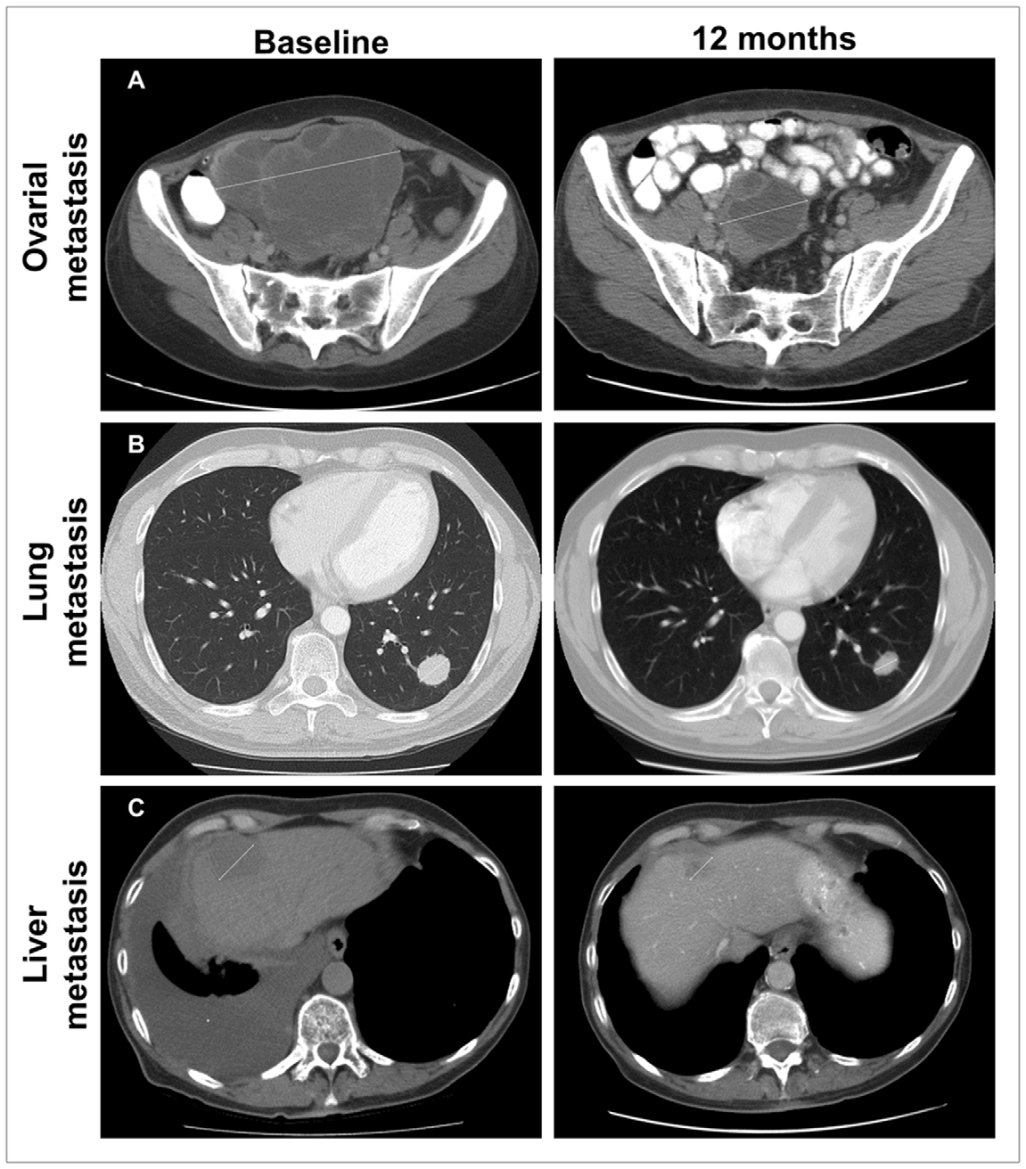
Computed tomography showing partial responses in three different patients at baseline and at 12 months. Panel A shows ovarian metastases in a 43 years old woman. Panel B shows lung metastases in a 50 years old man. Panel C shows liver metastases and pleural effusion (*) in a 70 years old man. Arrows show the largest diameter of the lesions.

In the study population of 35 patients, we observed 1 CR (3%), 5 PR (14%), and 5 SD >6 months (14%). Thus, 24 patients (69%) progressed on therapy, including three patients who progressed clinically before radiological tumor evaluation. Best overall response (BOR), measured as the change in the sum of largest diameter of the target lesions is illustrated in Figure 2A. Duration of the responses in relation to patient characteristics is illustrated in Figure 2B. Tumor responses were observed at metastatic sites such as skin, lymph nodes, lung, liver and ovaries (Fig 3).

**Figure 4:**
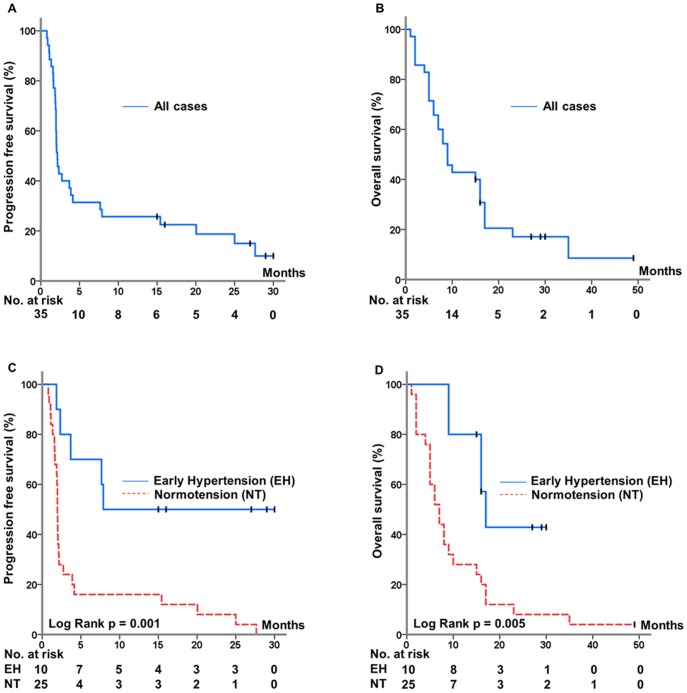
Kaplan Meyer plots of progression free survival (PFS) (A) and overall survival (OS) (B) in 35 metastatic melanoma patients treated with bevacizumab monotherapy. Early hypertension (EH) was significantly associated with PFS (C) and OS (D).

At 6 months of follow up, 11/35 (31)% of the patients had no sign of melanoma progression. This proportion was 8/20 for the first line patients and 3/15 for the second/third line patients, respectively. By August 2011, median PFS was 2.14 months whereas mean PFS was 7.7 months (range 0.8–30 months), with a median overall survival of 9 months (mean: 13, range: 1.1–49) (Fig 4 A and B). The median number of cycles was 4 (mean: 14, range: 1.0–64). No patients died of causes other than melanoma progression. Six of the patients are still alive, and 5 of them are still on bevacizumab treatment without signs of progression 15–30 months after starting bevacizumab.

Seven of 11 patients with DC developed early hypertension (EH) as defined by CTCAEv3.0. In contrast, only three of 24 (12.5%) patients with progressive disease (PD) developed EH (Chi-square test p<0.001). Median time to progression for patients who developed EH following bevacizumab treatment was 11.4 months compared to 2.0 months in normotensive patients. EH was significantly associated with prolonged PFS (log rank p = 0.001, Fig 4C) as well as improved overall survival (log rank p = 0.005, Fig 4D). To explore the possible association between the use of different antihypertensive drugs and disease progression we observed that 6 of 7 patients on concomitant beta blockers experienced DC following treatment with bevacizumab monotherapy. In comparison, 3/6 patients who used antihypertensive drugs other than beta blockers, or 2/22 who used no antihypertensive drugs experienced DC (Chi square test p<0.001).

Stage M1a and b disease was significantly associated with DC (7/7) as compared with M1c disease (4/28; Chi-square test p<0.001). Similarly, 9 of 14 patients with normal levels of lactate dehydrogenase (LDH) at baseline had DC as compared with 2 out of 21 with increased LDH (Chi-square test p = 0.001). No significant correlations were found between DC, OR, PFS or OS and *BRAF* or *NRAS* mutation status, performance status, sex or age.

### Safety

Bevacizumab monotherapy, given as 10 mg/kg q14d IV was in general well tolerated by the patients. No treatment related deaths were recorded. Treatment was stopped in two patients with CTCAE grade 4 toxicity (1 anaphylactic shock at cycle 2 and 1 lung embolus at cycle 5) and in one patient with grade 3 gastrointestinal toxicity (partial obstruction due to disease progression at cycle 1). Treatment was interrupted in one patient with grade 3 toxicity due to symptomatic left ventricular systolic dysfunction after 16 cycles. All adverse events are listed in Table S1 (online only). No dose reduction, interruption or postponement due to fatigue or hypertension was necessary and no hemorrhage was observed. Bevacizumab was delayed until proteinuria was <2 g/24 h in three patients (<4 weeks), but no treatment was stopped permanently due to proteinuria.

Hypertension according to CTCAEv3.0 was observed in 14 (40%) patients after initiation of bevacizumab. Grade 1∶4 (11%), grade 2∶3 (9%) and grade 3∶7 (20%), respectively. Median time to induced hypertension was 43 days (mean: 59, range: 27–239). We defined early hypertension (EH) as hypertension ≥ grade I occurring before 1^st^ tumor response evaluation at 8 weeks. EH was recorded in 10 patients whereas 4 patients developed hypertension at a later time point. As listed in Table 1, 8 (23%) of the patients were treated for hypertension at the time of inclusion. Type of antihypertensive drugs used is listed in Table S2.

## Discussion

While some phase II studies have evaluated the use of bevacizumab in concert with interferon alpha 2B [11], [28], interferon alpha 2A [12] or chemotherapy [13], [14], to the best of our knowledge this is the first study evaluating bevacizumab monotherapy in metastatic malignant melanoma. Our results provide a proof-of-principle that bevacizumab monotherapy is active in metastatic melanoma with a disease control rate of 31% and a 6 months PFS rate of 31%. In consistency with our finding, a recently published study of the multi kinase inhibitor axitinib (including VEGF receptor 1, 2 and 3) given as monotherapy showed a OR rate of 18.8% and a DC rate of 37.5% in a similar patient population [29]. These results are strikingly in line with ours and are in support of a significant subgroup of melanoma patients being susceptible to anti-VEGF strategies. Although not meeting the primary objective of increased median progression free survival, a recently published placebo controlled randomized phase II study showed encouraging overall survival data in metastatic melanoma patients treated with carboplatin and paclitaxel ± bevacizumab [15]. In contrast to our present findings, the patients who benefitted most from that combination were those with increased LDH and M1C disease, possibly indicating different sensitivity between patients groups to combination therapy and monotherapy.

In metastatic melanoma new treatment options have recently emerged targeting *BRAF* [5] or CTLA-4 [4] showing improved overall survival, but these treatments are associated with significant toxicities and costs. In addition, for *BRAF* negative patients or patients with non-immunogenic disease only limited effective treatment options are available. Significantly, in our study there was a subset of patients (14%) showing long-term survival on treatment (>2 years), independent of *BRAF* or *NRAS* mutational status.

Predictive markers for response to antiangiogenic treatment are urgently needed to guide clinical decision making and to target therapy towards well selected subgroups of patients. The present lack of useful predictive biomarkers decrease the likelihood of benefits, cost-effectiveness and therapeutic outcomes [14], [30]. We provide evidence that the clinical benefit of bevacizumab monotherapy in metastatic melanoma is almost exclusively limited to those patients who develop early hypertension during treatment. This can in part be explained by the fact that some non-responders did not have sufficient time on bevacizumab to develop hypertension. Still, most of the hypertensive patients (10/14) were recorded with hypertension before the 1^st^ tumor evaluation in week 8 (early hypertension). This phenomenon has been reported for several antiangiogenic drugs [30], and early onset hypertension is one of few markers at the present have been found to predict response to antiangiogenic drugs [30], [31].

The causal mechanism behind induced hypertension by antiangiogenic drugs is still elusive. VEGF upregulates nitric oxide [32] and prostacyclin [33], leading to vasodilatation, which is counteracted by bevacizumab. Also, the secondary hypotension following vascular permeability and leakiness caused by VEGF is counteracted by VEGF inhibition [34]. The angiogenic effect of players in the sympathetic nervous system associated with hypertension like norepinephrine (NE), has been reported [35]. Induction of VEGF and HIF-1α expression by NE was completely abolished by the beta blocker propranolol [36], suggesting a possible dual inhibition of VEGF when beta blockers are given together with bevacizumab. Clinical impact of beta blockers in cancer patients has been the focus of several large clinical and epidemiological studies, and these drugs can significantly reduce cancer progression and mortality [37]–[40], and might represent a promising drug combination with bevacizumab. Interestingly therefore, we found beta blocker use together with bevacizumab to be significantly associated with disease control. Still, this trial was not designed to analyze beta blocker use independently from hypertension, and the data must be interpreted with caution. [41]

In conclusion, bevacizumab monotherapy yielded promising data regarding disease control, progression free survival and overall survival in patients with metastatic melanoma, and the responders were typically characterized by induced hypertension early during therapy.

## Supporting Information

Table S1 Drug related toxicities of bevacizumab 10 mg/kg q2w for metastatic melanoma (n = 35). NCI CTCAE v3.0*.
(DOC)

Table S2 **Antihypertensive drugs used during treatment in 35 patients.**
(DOC)

Flow Diagram S1 **CONSORT 2010 Flow Diagram.**
(DOC)

Protocol S1 **Trial Protocol.**
(DOC)
