# Supplementary material for: Clinical Efficacy and Safety of Bevacizumab Monotherapy in Patients with Metastatic Melanoma: Predictive Importance of Induced Early Hypertension
Source: PLoS One. 2012 Jun 15;7(6):e38364. doi: 10.1371/journal.pone.0038364 (PMC3376108; doi:10.1371/journal.pone.0038364)
Supplement: Table S1 — Drug related toxicities of bevacizumab 10 mg/kg q2w for metastatic melanoma (n = 35). NCI CTCAE v3.0*. (DOC) [file pone.0038364.s001.doc]

## Supplemental Table S1

| **Table S1. Drug related toxicities of bevacizumab 10mg/kg q2w for metastatic melanoma (n=35). NCI CTCAE v3.0*.** | | | | | | | | | | |
| --- | --- | --- | --- | --- | --- | --- | --- | --- | --- | --- |
|  | All grades | | Grade 1 | | Grade 2 | | Grade 3 | | Grade 4 | |
| **Adverse effect** | No. | % | No. | % | No. | % | No. | % | No. | % |
| Fatigue | 5 | 14 | 3 | 9 | 2 | 6 |  |  |  |  |
| Proteinuria | 12 | 34 | 9 | 26 | 3 | 9 |  |  |  |  |
| Pain | 3 | 9 | 3 | 9 |  |  |  |  |  |  |
| Ileus | 1 | 3 |  |  |  |  | 1 | 3 |  |  |
| Left ventricular  systolic dysfunction | 1 | 3 |  |  |  |  | 1 | 3 |  |  |
| Allergy | 1 | 3 |  |  |  |  |  |  | 1 | 3 |
| Nausea | 2 | 6 | 2 | 6 |  |  |  |  |  |  |
| Lung embolus | 1 | 3 |  |  |  |  |  |  | 1 | 3 |
| Hypertension | 14 | 40 | 4 | 11 | 3 | 9 | 7 | 20 |  |  |
| * National Cancer Institute Common Terminology Criteria for Adverse Events v.3.0. | | | | | | | | | | |
