# Supplementary material for: Clinical Efficacy and Safety of Bevacizumab Monotherapy in Patients with Metastatic Melanoma: Predictive Importance of Induced Early Hypertension
Source: PLoS One. 2012 Jun 15;7(6):e38364. doi: 10.1371/journal.pone.0038364 (PMC3376108; doi:10.1371/journal.pone.0038364)
Supplement: Table S2 — Antihypertensive drugs used during treatment in 35 patients. (DOC) [file pone.0038364.s002.doc]

## Supplemental Table S2

| **Table S2. Antihypertensive drugs used during treatment in 35 patients** | | |
| --- | --- | --- |
| **Drug type** | No. | % |
| β1-selective beta blockers | 5 | 14 |
| Non-selective beta blockers | 2 | 6 |
| ACE* inhibitors | 2 | 6 |
| AT II** antagonists | 8 | 23 |
| Calcium antagonists | 7 | 20 |
| Diuretics | 7 | 20 |
| No antihypertensive drugs | 22 | 62 |
| Combinations with beta blockers | 5 | 14 |
| Combinations without beta blockers | 6 | 17 |
| Beta blockers *** without combination | 2 | 6 |
| * Angiotensin converting enzyme. ** Angiotensin II.  *** β1-selective beta blockers | | |
