# Supplementary material for: Clinical Efficacy and Safety of Bevacizumab Monotherapy in Patients with Metastatic Melanoma: Predictive Importance of Induced Early Hypertension
Source: PLoS One. 2012 Jun 15;7(6):e38364. doi: 10.1371/journal.pone.0038364 (PMC3376108; doi:10.1371/journal.pone.0038364)
Supplement: Flow Diagram S1 — CONSORT 2010 Flow Diagram. (DOC) [file pone.0038364.s003.doc]

**CONSORT 2010 Flow Diagram**

**Allocation**

**Analysis**

**Follow-Up**

**Enrollment**

Assessed for eligibility (n= 52 )

Excluded (n= 17 )

♦  Not meeting inclusion criteria (n= 16)

♦  Declined to participate (n= 1 )

Analysed (n= 35 )
♦ Excluded from analysis (give reasons) (n= 0 )

Lost to follow-up (give reasons) (n= 0)

Discontinued intervention (give reasons) (n= 3 )

Allocated to intervention (n= 35 )

♦ Received allocated intervention (n= 35 )

♦ Did not receive allocated intervention (give reasons) (n= 0 )
