# Supplementary material for: Clinical Efficacy and Safety of Bevacizumab Monotherapy in Patients with Metastatic Melanoma: Predictive Importance of Induced Early Hypertension
Source: PLoS One. 2012 Jun 15;7(6):e38364. doi: 10.1371/journal.pone.0038364 (PMC3376108; doi:10.1371/journal.pone.0038364)
Supplement: Protocol S1 — Trial Protocol. (DOC) [file pone.0038364.s004.doc]

**Protocol title:**

**Efficacy of bevacizumab monotherapy in treatment of metastatic melanoma and predictive value of angiogenic markers.**

**Protocol identification number: 94070**

| *12/4-05* | **Protocol approval** |
| --- | --- |
|  |  |

Version: 12.04.05

Contact addresses

| **Study coordinator:** | ODDBJORN STRAUME, MD PhD  Department of Oncology  Haukeland University Hospital  5021 Bergen  Norway Phone: +47 55975000, +47 55973160 Fax: +47 55973158, +4755972046 e-mail: [oddbjorn.straume@gades.uib.no](mailto:oddbjorn.straume@gades.uib.no) |
| --- | --- |

Table of contents:

1 Background and introduction [4](#__RefHeading___Toc90533710)

1.1 Background Disease Information [4](#__RefHeading___Toc90533711)

1.2 Background Therapeutic Information [5](#__RefHeading___Toc90533712)

2 Objectives of the trial [8](#__RefHeading___Toc90533713)

2.1 Primary objective [8](#__RefHeading___Toc90533714)

2.2 Secondary Objectives [8](#__RefHeading___Toc90533715)

2.3 End-points [8](#__RefHeading___Toc90533716)

3 Patient selection criteria [9](#__RefHeading___Toc90533717)

4 Trial Design [9](#__RefHeading___Toc90533718)

4.1 Treatment Plan [9](#__RefHeading___Toc90533719)

4.1.1 Drug Administration [9](#__RefHeading___Toc90533720)

4.1.2 Patient Monitoring [9](#__RefHeading___Toc90533721)

4.1.3 Dose Adjustments [9](#__RefHeading___Toc90533722)

4.1.4 Duration of Therapy [9](#__RefHeading___Toc90533723)

4.1.5 Concomitant therapy [9](#__RefHeading___Toc90533724)

5 Clinical evaluation, laboratory tests, follow-up [9](#__RefHeading___Toc90533725)

5.1 Before treatment start [9](#__RefHeading___Toc90533726)

5.2 During treatment [9](#__RefHeading___Toc90533727)

5.3 After the end of treatment (Follow-up) [9](#__RefHeading___Toc90533728)

5.4 Summary table [9](#__RefHeading___Toc90533729)

6 Criteria of evaluation [9](#__RefHeading___Toc90533730)

7 Statistical design [9](#__RefHeading___Toc90533731)

7.1 Sample size [9](#__RefHeading___Toc90533732)

7.2 Statistical Analysis [9](#__RefHeading___Toc90533733)

8 Translational research [9](#__RefHeading___Toc90533734)

9 Patient registration [9](#__RefHeading___Toc90533735)

10 Forms and procedures for collecting data [9](#__RefHeading___Toc90533736)

11 Reporting adverse events [9](#__RefHeading___Toc90533737)

11.1 Definitions [9](#__RefHeading___Toc90533738)

11.2 Reporting procedure [9](#__RefHeading___Toc90533739)

12 Ethical considerations [9](#__RefHeading___Toc90533740)

12.1 Informed consent [9](#__RefHeading___Toc90533741)

13 Trial insurance [9](#__RefHeading___Toc90533742)

14 Publication policy [9](#__RefHeading___Toc90533743)

Table of appendices:

Appendix A: References [9](#__RefHeading___Toc73067108)

Appendix B: Patient information sheets 29

# Background and introduction

## Background Disease Information

In Norway, cutaneous malignant melanoma is the second most frequent and the most frequent cancer type in middle-aged (30-54 years) females and males, respectively, and the incidence has six-doubled during the last 30 years. The age adjusted incidence rate in 2001 was 16.0/100,000 for females and 14/100,000 for males (Cancer Registry of Norway, [*http://www.kreftregisteret.no/*](http://www.kreftregisteret.no/)). The 5 year survival rate for all stages is 80% and 90% for men and women respectively, whereas for patients with distant metastases these numbers are 20% and 15%. Median survival for patients with metastatic melanoma is 6 months.

Many agents have been investigated for anti-tumor effect in melanoma, but there is no accepted standard therapy. Biochemotherapy, combining cytotoxic drugs with Interleukin-2 or Interferon alpha, has not been shown to be superior to single agent Dacarbazine (DTIC)(Lens & Eisen, 2003), which is regarded to be the most active agent. Other biological approaches like vaccination are currently under investigation(Lawson, 2004), but still no efficient treatment for metastatic melanoma is available. DTIC induces objective remission in 20% of the patients, but without significant impact on survival. A recent study on xenograft models has even suggested that DTIC might increase tumor growth in resistant tumors by inducing an activation of growth factors like VEGF and IL-8(Lev et al., 2004; Lev et al., 2003).

Of the identified angiogenic factors, vascular endothelial growth factor VEGF is the most potent and specific and has been identified as a crucial regulator of both normal and pathologic angiogenesis(Ferrara & Davis-Smyth, 1997). VEGF is a highly conserved, homodimeric, secreted, heparin-binding glycoprotein. VEGF produces a number of biologic effects, including endothelial cell mitogenesis and migration, induction of proteinases leading to remodeling of the extracellular matrix, increased vascular permeability and maintenance of survival for newly formed blood vessels(Ferrara & Davis-Smyth, 1997). VEGF expression is regulated by hypoxia via molecular pathways similar to those regulating erythropoietin gene expression. The biologic effects of VEGF are mediated through binding and stimulation of two receptors on the surface of endothelial cells: Flt-1 (fms-like tyrosine kinase) and KDR (kinase domain region). Increased levels of VEGF expression have been found in most human tumors examined to date, including tumors of the lung, breast, thyroid, gastrointestinal tract, kidney, bladder, ovary, and cervix, as well as angiosarcomas and glioblastomas(Ferrara & Davis-Smyth, 1997).

Specifically in malignant melanoma, increased expression of VEGF has been associated with malignant progression(Erhard et al., 1997; Marcoval et al., 1997; Salven et al., 1997). In accordance with this, we found that practically all vertical growth phase melanomas express VEGF to some extent(Straume et al., 2000). Still, a recent experimental study indicated that the angiogenesis in poorly angiogenic melanomas was promoted solely by VEGF, whereas multiple angiogenic factors were involved in the angiogenesis of highly angiogenic melanomas(Rofstad & Halsor, 2000). This is supported by an experimental study showing a switch from dormant non metastatic vertical growth phase melanomas into progressively growing tumors by overexpression of VEGF. This switch was effectively blocked by a monoclonal anti VEGF antibody(Bayko et al., 1998).

Increasing evidence support the expression and functional importance of VEGF receptors in cell types other than endothelial cells(de Jong et al., 1998; Liu et al., 1995). VEGF receptors FLT-1 and KDR are present at various levels in tumor cells in most of the cases, and both receptors were significantly coexpressed with VEGF(Straume et al., 2000). This might suggest the presence of possible autocrine loops, and a significant association between KDR expression and tumor cell proliferation as estimated by Ki-67 staining in the subgroup of thicker tumors was reported(Straume et al., 2000). Some functional evidence has been published showing increased proliferation of KDR expressing cells(Dunk & Ahmed, 2001; Liu et al., 1995), or decreased proliferation of FLT-1 expressing cells(Dunk & Ahmed, 2001; Herold-Mende et al., 1999) in response to VEGF. The VEGF system might therefore be important for various processes involving other cell types including tumor cells.

Anti-angiogenic drugs, similar to direct acting cytotoxic drugs are most effective against the rapidly dividing tumor cells at the well oxygenated periphery of the tumor where angiogenesis occur most vigorously. On the other hand, vascular targeting agents (VTA) seem to be more active against vessels in the interior of a tumor, leading typically to widespread central necrosis(Thorpe, 2004). The addition of a cytotoxic drug with anti-vascular effect like vinblastine(Baguley et al., 1991; Eikesdal et al., 2001) might increase the anti-tumor response, and the anti-vascular effect might even be optimized by administering the drug more frequent (e.g. weekly) at levels below the maximum tolerated dose (MTD)(Bertolini et al., 2003; Hahnfeldt et al., 2003). This strategy may minimize the problems of host toxicity and acquired drug resistance. Also, there are studies suggesting that the treatment of melanoma patients with DTIC might be more effective by adding anti-VEGF agents(Lev et al., 2004; Lev et al., 2003).

We, and others have previously shown that angiogenesis plays an important role in melanoma progression(Srivastava et al., 1988; Straume et al., 2000; Streit et al., 1999). On this background, an anti-angiogenic strategy aiming to inhibit VEGF is justified. Furthermore, there has been increasing focus on the need of reliable biomarkers that are will predict response of new treatment strategies including anti angiogenic treatment(Chen, 2004; Hlatky et al., 2002; Lynch et al., 2004). An efficient selection of patients that are likely to respond to targeted therapies will be increasingly important in future cancer treatment, as the magnitude of treatment options increases along with the costs(Mayer, 2004).

The need of a new and effective treatment for the group of melanoma patients is urgently needed. This will be the first study to assess response rates of bevacizumab monotherapy in first line treatment of metastatic melanoma. In addition there will be a major focus on the identification of predictive biomarkers of bevacizumab efficacy.

## Background Therapeutic Information

**Bevacizumab, Anti-VEGF monoclonal antibody – rHuMAb VEGF**

A recombinant humanized version of a murine anti-human VEGF monoclonal antibody, named rhuMAb VEGF (bevacizumab), was designed to test the hypothesis that VEGF inhibition could clinically benefit cancer patients(Presta et al., 1997).

Inhibition of VEGF using an anti-VEGF monoclonal antibody blocks the growth of a number of human cancer cell lines in nude mice(Ferrara & Davis-Smyth, 1997). The human cancers represented by these cell lines that are growth-inhibited by anti-VEGF antibody include non-small cell lung cancer, colorectal cancer, breast cancer, prostate cancer, head and neck cancer, ovarian cancer and others. In addition, the combination of anti-VEGF antibody and chemotherapy in nude mice injected with human cancer xenografts resulted in an increased anti-tumor effect compared with antibody or chemotherapy treatment alone(Borgstrom et al., 1999). Preclinical studies on VEGF overexpressing xenografted melanoma cell lines, showed that melanoma progression could be effectively blocked by a monoclonal anti VEGF antibody(Bayko et al., 1998).

**Clinical studies- bevacizumab**

In a phase I study(Gordon et al., 2001) bevacizumab was administered as a single agent in subjects with advanced malignancies. This was an open-label, dose–escalation study evaluating initial safety and pharmacokinetics. Five dose levels (0.1 to 10 mg/kg) were evaluated. Patients received a total of four doses over 42-day treatment period. Another phase I study(Margolin et al., 2001) evaluated multiple doses of bevacizumab in combination with one of three cytotoxic chemotherapy regimens (5-FU/Leucovorin, carboplatin/paclitaxel, or doxorobucin) in patients with metastatic disease. Bevacizumab was administered as eight weekly doses of 3mg/kg. Bevacizumab was characterized by a slow clearance of ~ 3mL/kg/day and a terminal elimination half-life of ~20 days. Co-administration of bevacizumab did not appear to result in a change in the systemic concentrations of the cytotoxic agents. Three events of tumor related bleeding were observed (3 of 25 treated patients)(Gordon et al., 2001). Bevacizumab was in general well tolerated with, though grade 1-2 toxicities including asthenia, headache and nausea were recorded(Fernando & Hurwitz, 2004; Gordon et al., 2001; Margolin et al., 2001).

In phase II studies, bevacizumab was administered either as single agent or in combination with cytotoxic chemotherapy in several tumor types(Bergsland & Dickler, 2004; Chen, 2004; Johnson et al., 2004; Kabbinavar et al., 2003). The most important safety issue was hemorrhagic events. Six out of 66 bevacizumab treated patients with NSCLC had life-threatening hemoptysis or hematemesis. Four of these events were fatal, and these were all of squamous cell histology(Johnson et al., 2004). Other bevacizumab associated side effects reported are venous and arterial thromboses. Asymptomatic proteinuria without evidence of renal dysfunction and hypertension have frequently been reported(Bergsland & Dickler, 2004). Less clinically severe events are epistaxis, diarrhea, fever, rash, and headache have been seen in all clinical trials to date(Bergsland & Dickler, 2004; Kabbinavar et al., 2003).

Clinical experience with bevacizumab has demonstrated that the agent is biologically active(Chen, 2004). In a randomized phase II study in patients with metastatic renal cell carcinoma, bevacizumab monotherapy significantly prolonged time to progression(Yang et al., 2003). Also in breast cancer, bevacizumab monotherapy has increased response rates(Cobleigh et al., 2003). In colorectal cancer, the combination therapy of bevacizumab and irinotecan/5FU was associated with a significantly improved survival when compared with irinotecan/5FU alone(Hurwitz et al., 2004). The main toxicities observed were thrombosis, proteinuria, hypertension, fever, chills, rash, headache, infection, epistaxis, and mouth ulceration(Fernando & Hurwitz, 2004).

There are several ongoing studies of bevacizumab(Chen, 2004). In malignant melanoma one study combines bevacizumab with IFN alpha and another combines with imatinib. These studies are still including patients. In a study of metastatic breast cancer, bevacizumab was combined with vinorelbine(Burstein et al., 2002; Rugo, 2004), a vinca alkaloid similar to vinblastine. Bevacizumab with vinorelbine was well tolerated and only minor occurrences of hypertension, proteinuria and epistaxis were seen. No major bleeding events or thrombotic events were observed, and other side effects were consistent with known side effects by the use of vinorelbine.

*Pharmaceutical Data*

*Supplied: Roche Norway*

*Stability: Opened vials must be used within 8 hours. Once bevacizumab has been added to sterile saline, the solution must be used within 8 hours,*

*Storage: Refrigerated at 2°C-8°C*

*Solution Preparation: For administration, bevacizumab will be diluted in 0.9% Sodium Chloride Injection, to a total volume of 100 mL. Once bevacizumab/ placebo has been added to a bag of sterile saline, the solution must be administered within 8 hours.*

*Route of Administration: Intravenous infusion*

# Objectives of the trial

## Primary objective

- To determine the efficacy as measured by objective tumor response of first-line treatment of metastatic melanoma with bevacizumab monotherapy (every 14^th^ day).

## Secondary Objectives

- To explore relationships between measures of tumor expression of the molecular target and objective clinical response, and identify predictive markers
- To estimate the time to progression, survival and proportion of patients with stable disease.
- To examine the toxicity rate and toxicity profile of the drug given in schedule

## End-points

**Primary endpoint**: Clinical response rates (proportion of complete response and partial response) according to RECIST criteria

**Secondary endpoint**: Time to progression (TTP)

Overall survival (OS)

Safety data

Please refer to Section 7 “Criteria of evaluation”

# Patient selection criteria

- Histologically confirmed metastatic (unresectable) melanoma and with progressive disease

**LEVEL A (second line)**: after confirmed progression on standard first line treatment with dacarbazine.

**LEVEL B (first line)**: when objective clinical response is observed in LEVEL A, patients will be included for first line treatment with bevacizumab.

- Have a WHO performance status 0-2
- Age >18 years,
- No pregnant or lactating patients can be included.
- Able to undergo outpatient treatment
- Patients must have clinically and/or radiographically documented measurable disease according to RECIST criteria. At least one site of disease must be unidimensionally measurable as follows:

X-ray or physical exam > 20 mm

Spiral CT scan > 10 mm

Non-spiral CT scan > 20 mm

All radiology studies must be performed within 28 days prior to registration (35 days if negative).

- At least 4 weeks since adjuvant interferon alpha
- No prior interferon alpha or IL-2 for metastatic disease
- No more than 1 prior chemotherapy regimen for metastatic disease
- Recovered from prior chemotherapy
- Major surgical procedure or significant traumatic injury within 28 days prior to study treatment start. Biopsy or fine needle aspiration within 5 days prior to study treatment start. Central venous line placement must be inserted at least 5 days prior to treatment start.
- No known brain metastases
- Minimum required laboratory data:

Hematology: absolute granulocytes > 1.0 x 10^9^/L

platelets > 100 x 10^9^/L

Biochemistry: bilirubin < 1.5 x upper normal limit

serum creatinine within normal limits

INR < 1.5

no clinical evidence of coagulopathy

- No symptomatic congestive heart failure
- No unstable angina pectoris
- No cardiac arrhythmia
- No history of thrombosis
- No full-dose oral coumarin-derived anticoagulants (INR>1.5) or heparin, thrombolytic agents, or chronic, daily treatment with aspirin (>325 mg/day).
- No non-steroidal anti-inflammatory medications (those known to inhibit platelet function at doses used to treat chronic inflammatory diseases).
- No uncontrolled hypertension
- Absence of any psychological, familial, sociological or geographical condition potentially hampering compliance with the study protocol and follow-up schedule; those conditions should be discussed with the patient before registration in the trial
- Before patient registration/randomization, written informed consent must be given according to national and local regulations.

# Trial Design

This is a non randomized, non blinded, single center Phase II study, taking place at the Haukeland University Hospital, Bergen Norway. For statistical design and calculation of sample size, please refer to section 8.1. The design for phase II clinical trials as proposed by Simon(Simon, 1993) is used to assess the efficacy of bevacizumab monotherapy in metastatic malignant melanoma. Accordingly, patients will be enrolled in two steps. The primary endpoint is the clinical response rate defined as complete response (CR) + partial response (PR) according to RECIST criteria. Each treatment cycle consist of infusion bevacizumab on day 1. Day 15 is day 1 of the next cycle. After every 2nd cycle toxicity will be assessed, and after every 4 cycles response rates will be evaluated. Patients are planned to receive 12 cycles of protocol treatment.

According to ethical principles, patients will initially be included after confirmed progression on standard first line treatment with dacarbazine (LEVEL A). When clinical and objective response is observed on bevacizumab monotherapy in LEVEL A, all new patients will subsequently be enrolled for first line bevacizumab for metastatic melanoma (LEVEL B). Patients included at LEVEL B with disease progression on bevacizumab monotherapy will be offered standard treatment dacarbazine in second line.

Medical parameters will be collected to correlate with clinical outcome. In addition, tissue samples and blood samples will be acquired at baseline and every 8 weeks for biomarker analysis (section 8). These biomarkers will be examined and their predictive value of clinical outcome will be studied.

## Treatment Plan

Patients with metastatic melanoma meeting the inclusion criteria will be given bevacizumab according to the following dose schedule. One cycle consist of infusion of bevacizumab on day 1. Day 15 is day 1 of the next cycle. In other words, a new cycle starts every 2 weeks. Protocol treatment includes 12 cycles, as outlined in section 4.1.4.

### Drug Administration

Bevacizumab (B) 10 mg/kg is given as intravenous infusion, dissolved in a total volume of 100 mL of 0.9% Sodium Chloride Injection, over 90 minutes, if no infusion associated adverse effects occur, the next dose can be given in 60 minutes, and the next in 30 minutes.

### Patient Monitoring

Blood pressure and heart rate are to be assessed every 30 minutes during the infusion of bevacizumab at cycle1and 2. If no changes are seen, then end of infusion assessment only will be required for subsequent cycles

### Dose Adjustments

Adverse events will be graded using the NCI Common Terminology Criteria for Adverse Events Version 3.0 (CTCAE) ([http://ctep.cancer.gov/reporting/ctc.html](http://ctep.cancer.gov/forms/CTCAEv3.pdf)). (A version can also be found on the server together with the MelanomaCRF database)

The major adverse effects of bevacizumab which limit dose is/are:

- Thromobembolism grade 3
- Haemorrhage grade 2

If side effects occur which is not contained in the NCI-CTCAE, the four-point scale (mild, moderate, severe, life-threatening) below will be used.

Mild: discomfort noticed but no disruption of normal daily activity.

Moderate: discomfort sufficient to reduce or affect daily activity.

Severe: inability to work or perform normal daily activity

Life Threatening: represents an immediate threat to life

Life threatening toxicities seen with bevacizumab to date have been hemorrhage, thrombosis and gastro-intestinal perforation. Less severe toxicities include proteinuria, hypertension, fever, chills, rash, headache, infection, epistaxis and mouth ulceration. Because of the long terminal half-life of bevacizumab (20 days) the discontinuation of treatment in case of an adverse events is not expected to influence its clinical evolution and therefore, the management of adverse events is based on institution of adequate treatment. There will be no dose modifications of bevacizumab during the study unless the patients by > 10% weight loss.

Schedule modifications by thrombosis due to bevacizumab

| Grade of Event | Management/Next Dose |
| --- | --- |
| grade 3 | Hold bevacizumab until patient is on a stable dose of anticoagulant |
| grade 4 | Off protocol therapy |
| * Patients requiring a delay of > 4 weeks should go off protocol therapy. | |

**Schedule modifications by hemorrhage due to bevacizumab**

| Grade of Event | Management/Next Dose |
| --- | --- |
| < grade 1 | Symptomatic – no change in dose |
| grade 2 | Symptomatic – no change in dose |
| grade 3 | Discontinue the treatment with bevacizumab |
| grade 4 | Discontinue the treatment with bevacizumab |
| * Patients requiring a delay of > 4 weeks should go off protocol therapy. | |

**Schedule modifications by proteinuria due to bevacizumab**

| Grade of Event | Management/Next Dose |
| --- | --- |
| > 2g/24hr | Hold bevacizumab until proteinuria improves to ≤ 2g/24hr |
| grade 4 | Off protocol therapy |
| * Patients requiring a delay of > 4 weeks should go off protocol therapy. | |

**Schedule modifications by hypertension due to bevacizumab**

| Grade of Event | Management/Next Dose |
| --- | --- |
| grade 3 | If not controlled by medication, discontinue bevacizumab |
| grade 4 | Discontinue the treatment with bevacizumab |
| * Patients requiring a delay of > 4 weeks should go off protocol therapy. | |

**Schedule modifications by gastrointestinal perforation due to bevacizumab**

| Grade of Event | Management/Next Dose |
| --- | --- |
| Gastrointestinal perforation | Discontinue treatment with bevacizumab |

### Duration of Therapy

The duration of the therapy is defined as 12 cycles (24 weeks) of treatment, and this will be the end of protocol therapy, but actual duration of treatment will depend on individual response and tolerance (see 5.3). During this Post-Study Treatment Phase, assessments include survival, tumor response and serious adverse events.

Patients with progressive disease (see definition Section 6.0) must be taken off treatment at time of progression, and patients included at LEVEL 2 will be offered second line dacarbazine. Patients who are responding (complete or partial), or whose disease is stable, will be treated until progression of disease, intolerable toxicity or patient refusal to continue with the study. Patients may also discontinue protocol therapy by intercurrent illness, which would, in the investigators judgement affect patients’ safety, the ability to deliver treatment or the primary study endpoints.

### Concomitant therapy

At study initiation, patients should continue with their concomitant medications, as directed by their physician.The most severe toxicities seen with bevacizumab to date have been hemorrhage, thrombosis and gastro-intestinal perforation(Fernando & Hurwitz, 2004). For this reason, the use of the following agents is limited. Full-dose oral coumarin-derived anticoagulants (INR>1.5) or heparin, thrombolytic agents, or chronic, daily treatment with aspirin (>325 mg/day) or non-steroidal anti-inflammatory medications (those known to inhibit platelet function at doses used to treat chronic inflammatory diseases) are excluded at entry into the study. Low-dose or heparin is permitted, as is low-dose aspirin (<325 mg/day), occasional use of non-steroidal anti-inflammatory medication, or regular use of nonsteroidal anti-inflammatory medication of the kind known not to inhibit platelet function.

Other anti-cancer drug are not allowed. Palliative radiation therapy, directed against lesions other than the target lesions for evaluation, is allowed and should be recorded in the CRF. Supportive or other palliative measures are allowed.

# Clinical evaluation, laboratory tests, follow-up

## Before treatment start

Patients must have clinically and/or radiographically documented measurable disease according to RECIST criteria. At least one site of disease must be unidimensionally measurable as follows:

X-ray, physical exam > 20 mm

Spiral CT scan > 10 mm

Non-spiral CT scan > 20 mm

All radiology studies, including dynamic contrast enhanced MRI (DCE MRI), must be performed within 28 days prior to registration (35 days if negative). All biochemistry analyses must be performed within 1 week prior to registration. As summarized in table 6.4 the baseline investigations include a physical examination, blood samples for hematology and biochemistry as well as for flow-cytometri and s-VEGF by ELISA, and similarly for urine samples. Baseline measures of the target lesions by radiology must be undertaken. And also the baseline measures of tumor blood flow by DCE MRI. If a biopsy of target lesions was not done after evidence of progression on first line treatment, a biopsy from target lesion will be performed at baseline to be subjected to translational studies as outlined in section 8.

## During treatment

Physical examination of the patient as well as routine blood sampling, will be performed before every new course of the treatment. Adverse events will be recorded before every new treatment. Response evaluation will be performed after every 4^th^ cycle and includes physical examination, hematology, biochemistry, radiology, target lesion biopsy (only by progression), dynamic MRI and peripheral blood for translational research studies.

## After the end of treatment (Follow-up)

After 12 cycles the protocol treatment ends. Patients with response or stable disease after initial response will be offered to continue treatment until progressive disease or intolerable toxicity. These patients will continue following the same schedule as by the 1^st^ 12 cycles.

After progression patients will be followed by physical examination every 4^th^ week, to assess late toxic effects. Survival data will be collected.

## Summary table

| Cycle |  | I | II | III | IV |  | V |
| --- | --- | --- | --- | --- | --- | --- | --- |
| Required Investigations | Prestudy | Day 1 | Day 15 | Day 29 | Day 43 | Day 54-55 | Day  57 |
| Physical | x | x | x | x | x | x | x |
| Hematology | x | x | x | x | x | x | x |
| Biochemistry | x | x | x | x | x | x | x |
| Radiology^1^ | x |  |  |  |  | x |  |
| Other Investigations |  |  |  |  |  |  |  |
| Biopsy of target lesion | x |  |  |  |  | x^2^ |  |
| Dynamic MRI | x |  |  |  |  | x |  |
| Adverse Events |  |  | x | x | x |  | x |
| Peripheral blood for research. | x |  |  |  |  | x |  |
| 1. Patients with a CR or PR should have scans repeated after 4 weeks to confirm response. 2. Only by progressive disease | | | | | | | |

# Criteria of evaluation

Definitions

Evaluable for response. All eligible patients will be included in the response rate calculation. The subset that will be assigned a response category (CR, PR, SD or PD; see definitions below) are all patients who have received at least one treatment and have their disease re-evaluated. Patients on protocol will have their response classified according to the definitions set out below(Duffaud & Therasse, 2000).

Response and Evaluation Endpoints

Response and progression will be evaluated in this study using the new international criteria proposed by the RECIST (Response Evaluation Criteria in Solid Tumors) committee(Duffaud & Therasse, 2000). Changes in only the largest diameter (unidimensional measurement) of the tumor lesions are used in the RECIST criteria.

Measurable Disease.

Measurable lesions are defined as those that can be accurately measured in at least one dimension (longest diameter to be recorded) as > 20 mm with conventional techniques (physical examination, CT, xray) or as > 10 mm with spiral CT scan. All tumor measurements must be recorded in millimetres.

Non-measurable Disease.

All other lesions (or sites of disease), including small lesions (longest diameter < 20 mm with conventional techniques or < 10 mm with spiral CT scan) are considered non-measurable disease. Bone lesions, leptomeningeal disease, ascites, pleural/pericardial effusions, lymphangitis cutis/pulmonis, inflammatory breast disease, abdominal masses (not followed by CT or MRI) and cystic lesions are all non-measurable.

Target Lesions.

All measurable lesions up to a maximum of 5 lesions per organ and 10 lesions in total representative of all involved organs should be identified as *target lesions* and be recorded and measured at baseline. Target lesions should be selected on the basis of their size (lesions with the longest diameter) and their suitability for accurate repetitive measurements (either by imaging techniques or clinically). A sum of the longest diameter (LD) for *all target lesions* will be calculated and reported as the baseline sum LD. The baseline sum LD will be used as reference to further characterize the objective tumor response of the measurable dimension of the disease. If there are > 10 measurable lesions, those not selected as *target lesions* will be considered together with non-measurable disease as *non-target lesions* (see 10.2.4).

Non-target Lesions.

All non-measurable lesions (or sites of disease) plus any measurable lesions over and above the 10 listed as *target lesions*. Measurements are not required but these lesions should be noted at baseline and should be followed as “present” or “absent”.

RESPONSE

All patients will have their BEST RESPONSE on study classified as outlined below:

Complete Response (CR): disappearance of all clinical and radiological evidence of tumor (both *target* and *non-target*).

Partial Response (PR): at least a 30% decrease in the sum of LD of target lesions, taking as reference the baseline sum LD.

Stable Disease (SD): steady state of disease. Neither sufficient shrinkage to qualify for PR nor sufficient increase to qualify for PD. Stable disease will be classified as response to treatment only if duration > 4 months.

PROGRESSION

Progressive Disease (PD): at least a 20% increase in the sum of LD of measured lesions taking as references the smallest sum LD recorded since the treatment started. Appearance of new lesions will also constitute progressive disease. In exceptional circumstances, unequivocal progression of non-target lesions may be accepted as evidence of disease progression.

| Target Lesions | Non-Target Lesions | New Lesions | Overall Response | Best Response for this category also requires |
| --- | --- | --- | --- | --- |
| CR | CR | No | CR | > 4 wks. confirmation |
| CR | Non-CR/Non-PD | No | PR | > 4 wks. confirmation |
| PR | Non-PD | No | PR |
| SD | Non-PD | No | SD | documented at least once > 4 wks. from baseline |
| PD | Any | Yes or No | PD | no prior SD, PR or CR |
| Any | PD* | Yes or No | PD |
| Any | Any | Yes | PD |
| * In exceptional circumstances, unequivocal progression in non-target lesions may be accepted as disease progression.  Note:  Patients with a global deterioration of health status requiring discontinuation of treatment without objective evidence of disease progression at that time should be reported as “*symptomatic deterioration”*. Every effort should be made to document the objective progression even after discontinuation of treatment. | | | | |

Response Duration

Response duration will be measured from the time measurement criteria for CR/PR (whichever is first recorded) are first met until the first date that recurrent or progressive disease is objectively documented, taking as reference the smallest measurements recorded since the treatment started.

Stable Disease Duration

Stable disease duration will be measured from the time of start of therapy until the criteria for progression are met, taking as reference the smallest measurements recorded since the treatment started.

Methods of Measurement

The same method of assessment and the same technique should be used to characterize each identified and reported lesion at baseline and during follow-up.

Clinical Lesions.

Clinical lesions will only be considered measurable when they are superficial (e.g. skin nodules, palpable lymph nodes). For the case of skin lesions, documentation by color photography including a ruler to estimate the size of the lesion is recommended.

Chest X-ray.

Lesions on chest X-ray are acceptable as measurable lesions when they are clearly defined and surrounded by aerated lung. However, CT is preferable. CT, MRI. CT and MRI might be the best currently available and reproducible methods to measure target lesions selected for response assessment. Conventional CT and MRI should be performed with cuts of 10 mm or less in slice thickness contiguously. Spiral CT should be performed using a 5 mm contiguous reconstruction algorithm. This applies to the chest, abdomen and pelvis. Head & neck and extremities usually require specific protocols.

Ultrasound.

When the primary endpoint of the study is objective response evaluation, ultrasound (US) should not be used to measure tumor lesions that are clinically not easily accessible. It is a possible alternative to clinical measurements for superficial palpable nodes, subcutaneous lesions and thyroid nodules. US might also be useful to confirm the complete disappearance of superficial lesions usually assessed by clinical examination.

Tumor Markers.

Tumor markers alone cannot be used to assess response. If markers are initially above the upper normal limit, they must normalize for a patient to be considered in complete clinical response.

# Statistical design

## Sample size

**Non randomized, non blinded, single center single arm, two stage, phase II study.**

The optimal two-stage design for phase II clinical trials proposed by Simon (ref.) is used to assess the efficacy of bevacizumab in metastatic malignant melanoma. The primary endpoint is the clinical response rate defined as complete response (CR) + partial response (PR) according to RECIST criteria. It is assumed that the new regimen will have a target response rate of 30%. A response rate of 10% or lower is considered no better than the standard first-line therapy and regimen will be rejected under this circumstance. With 10% type I error rate and 90% power, Simon's design requires to enter 12 patients in the first stage. If 1 or less patients respond to the treatment, the trial will be stopped and the regimen will be declared as ineffective.

If there are 2 or more responses, 23 more patients will be entered in the study to reach a total of 35 patients. By the end of the study, the new regimen will be rejected if there are no more than 5 responses and will be considered efficacious otherwise. This design has the optimal property of minimizing the expected sample size under the null hypothesis. Under the null hypothesis, the probability of early stopping is 66% and the expected sample size is 19.84.

## Statistical Analysis

**Accrual and Duration of Study**

The estimated accrual for this study is 2-4 patients a month. Thus, patient accrual is expected to be completed within 12-18 months. Additional time is required to allow the response data to mature. All of the patients registered in the study will be accounted for. The number of patients who were not evaluable, who died or withdrew before treatment began will be specified. The distribution of follow-up time will be described and the number of patients lost to follow-up will be given.

**Safety Monitoring**

Adverse events will be monitored on an ongoing basis and their frequencies reported semi-annually. Toxic effects will be categorized using the NCI Common Terminology Criteria for Adverse Events, Version 3.0. The worst event for each patient will be described. Both events related and unrelated to treatment will be captured. Clinical and laboratory data will be tabulated and compared to normal ranges for the institution.

**Statistical analysis and statistical tests**

Descriptive statistics will be used to summarize the distribution of sosiodemographic variables, medical variables and angiogenic marker variables. Response rates and its 95% confidence interval estimates will be computed to assess clinical response rates. Chi-square test or Fishers exact test will be applied to examine the association between categorical variables. Two sample t-test and Wilcoxon rank-sum test will be used compare the distribution of continuous variables between two groups such as responders and non-responders.

Logistic regression analysis will be applied to examine the predictive effect of multiple prognostic variables. Kaplan-Meier estimates will be constructed for time-to-event Endpoints such as TTP and OS. Log rank-test will be applied for testing the difference of time-to-event endpoints between groups. Cox regression analysis will be used to examine the effect of multiple prognostic variables on time-to-event endpoint. Due to the small sample size and the nature of the phase II study, the above analyses will be considered exploratory in nature and the results need to be confirmed in future large-scale studies.

# Translational research

**Objectives of the translational research**

The need of predictive markers for response of treatment in malignant diseases has become more focused upon during the past decade(Ranson, 2004). Especially by anti angiogenesis and other targeted approaches the importance of biomarkers will become increasingly important(Folkman et al., 2001; Ruegg et al., 2003). By the implementation of prognostic markers, patients at high risk of recurrence may be selected for treatment, whereas predictive markers can select patients likely to respond to a specific treatment. In that way, patients that do not need treatment and patients that are unlikely to respond will not be subject to the treatment and its adverse effects. The objective of the translational part of the study is to identify biomarkers that can predict the efficacy of treatment with bevacizumab in metastatic malignant melanoma. All biological material will be handled and stored according to national guidelines.

**Angiogenic markers**

The most widely used method of assessing the angiogenic capacity is by counting immunostained microvessel in the most vascularized areas of the tumor (Hot Spots), and microvessel density (Weidner et al., 1991) are given as number of microvessel/m^2^. MVD is reported to be a significant prognostic marker in most kinds of human tumors(Weidner, 1998). Still, the predictive value of this biomarker has been questioned(Hlatky et al., 2002). There has been an intensive search for new angiogenic markers that might predict response on the tumor associated microvasculature or the tumor, but still no markers have been validated.

Endothelial cells proliferation as expressed by Ki-67 immunostaining has been shown to provide prognostic information in endometrial carcinoma (Stefanson & Akslen in press), and also apoptosis in endothelial cells might predict response to therapy(Hlatky et al., 2002). Also the presence of VEGF associated glomeruloid microvascular proliferations (GMPs) was shown to be associated with significantly impaired prognosis in melanoma, endometrial carcinoma, breast cancer and prostate cancer(Straume et al., 2002). This marker also might provide some predictive information on chemotherapy treatment of metastatic breast cancer (Akslen, in prep).

Specifically in malignant melanoma, increased expression of VEGF has been associated with malignant progression(Erhard et al., 1997; Marcoval et al., 1997; Salven et al., 1997). In accordance with this, we found that practically all vertical growth phase melanomas express VEGF to some extent(Straume & Akslen, 2001). Increasing evidence support the expression and functional importance of VEGF receptors in cell types other than endothelial cells(de Jong et al., 1998; Liu et al., 1995). VEGF receptors FLT-1 and KDR are present at various levels in tumor cells in most of the cases, and both receptors were significantly coexpressed with VEGF(Straume & Akslen, 2001). This might suggest the presence of possible autocrine loops, both the expression of VEGF and its receptors might be candidate predictive markers in anti VEGF strategies. Also, VEGF-C and VEGF-D and their respective receptors have been focused upon in melanoma, as well as lymphatic vessel density(Dadras et al., 2003; Shields et al., 2004; Straume & Akslen, 2002; Straume et al., 2003; Valencak et al., 2004)(Straume & Akslen BJC 2004).

Number of circulating endothelial cells, endothelial cell precursors as well as sVEGF by ELISA has been investigated for their predictive value of response to anti VEGF strategies(Willett et al., 2004), still the results are conflicting.

Dynamic contrast-enhanced magnetic resonance imaging is a candidate biomarker for assessing the effect of antiangiogenic treatment(Morgan et al., 2003), and is at this time maybe the best validated non invasive method of assessing vascularity of tumors.

**Analysis of biopsies**

These analyses will be performed at the Department of Pathology, The Gade Institute, Haukeland University hospital. Tissue from the primary tumor will be available as formaline fixed and paraffin embedded material. In an already ongoing study of chemorecistance in melanoma, biopsies from the metastatic lesions taken at the time of start of first line treatment with DTIC (LEVEL A) or bevacizumab (LEVEL B), as well as at the time of progression. This material will be available as snap frozen tissue as well as formaline fixed.

Some of the markers to be evaluated are:

- Microvessel density by immunohistochemistry
- (Lymphatic vessel density by immunohistochemistry)
- VEGF expression in tumor cells by immunohistochemistry
- VEGFR-2 (KDR) expression on endothelial cells and tumor cells by immunohistochemistry
- Proliferative rate by Ki-67 expression in endothelial cells and tumor cells by immunohistochemistry
- Apoptotic index Tunel test in endothelial cells and tumor cells by immunohistochemistry
- Presence of VEGF associated Glomeruloid Microvascular Proliferations

**Flow cytometri**

Before start of the treatment as well as after every 2 cycles (8 weeks) peripheral blood are taken from the patient for analysis by flow cytometri. These analyses will be performed at the department of hematology, Haukeland University hospital. Following markers are to be evaluated:

- The number of circulating endothelial cells in peripheral blood
- The number of endothelial cell precursors in peripheral blood
- S-VEGF by ELISA
- U- VEGF

- Circulating D-Dimer levels (Blackwell et al., 2004), thrombocytosis(Sorbye et al., 2004)

- Marker analysis in platelets

- Site of metastatic lesions. (It has been recently suggested that anti angiogenesis treatment is less efficient against lesions in heavily vascularized organs like liver, kidney and brain where vessel cooption might occur).

- Other

- Dynamic MRI. This procedure will be undertaken at startup as well as after every two cycles (8 weeks).

**Statistical analysis of biomarkers**

Correlations between discrete end point variables and discrete values of the candidate predictive values will be tested by Chi Square test. Analysis of correlation between continous and discrete values will be done by the Mann Whitney test. Time to progression between by the first line treatment of metastatic disease by DTIC will serve as an internal control in each patient and will be analyzed by the Spearman rank correlation test of paired data.

# Patient registration

Every patient referred to our center with metastatic melanoma meeting the inclusion criteria will be offered inclusion in the protocol. At the time of registration, the patient eligibility is checked by the responsible physician/ principal investigator. Before patient registration, written informed consent must be given according to national/ local regulations.

# Forms and procedures for collecting data

Data will be collected and stored according to national regulations. Data will be collected from the clinical examinations, the medical record, The Norwegian Cancer registry as well as Statistics Norway. The data will be associated with an anonymous case number and entered in an “electronic CRF” (Access database). The principal investigator will have access to all the different forms in the “electronic CRF” and will be in charge of the data handling, whereas study nurses will have access to forms relevant to thair specific data registration. See separate application to NSD.

# Reporting adverse events

## Definitions

An **Adverse Event (AE)** is defined as any untoward medical occurrence or experience in a patient or clinical investigation subject which occurs following the administration of the trial medication regardless of the dose or causal relationship. This can include any unfavorable and unintended signs (such as rash or enlarged liver), or symptoms (such as nausea or chest pain), an abnormal laboratory finding (including blood tests, x-rays or scans) or a disease temporarily associated with the use of the protocol treatment.

An **Adverse Drug Reaction (ADR)** is defined as any response to a medical product, that is noxious and/or unexpected, related to any dose.

**Response to a medicinal product** (used in the above definition) means that a causal relationship between the medicinal product and the adverse event is at least a reasonable possibility, i.e. the relationship cannot be ruled out.

An **Unexpected Adverse Drug Reaction** is any adverse reaction for which the nature or severity is not consistent with the applicable product information (e.g., Investigators’ Brochure).

A **Serious Adverse Event (SAE)** is defined as any undesirable experience occurring to a patient, whether or not considered related to the protocol treatment. A Serious Adverse Event (SAE) which is considered related to the protocol treatment is defined as a **Serious Adverse Drug Reaction (SADR)**.

Adverse events and adverse drug reactions which are considered as **serious** are those which result in:

- death
- a life threatening event (i.e. the patient was at immediate risk of death at the time the reaction was observed)
- hospitalization or prolongation of hospitalization
- persistent or significant disability/incapacity
- a congenital anomaly/birth defect
- any other medically important condition (i.e. important adverse reactions that are not immediately life threatening or do not result in death or hospitalization but may jeopardize the patient or may require intervention to prevent one of the other outcomes listed above)

## Reporting procedure

Any SEA occurring during study treatment will be reported by the principal investigator to SLV according to national laws and GCP.

# Ethical considerations

The responsible investigator will ensure that this study is conducted in agreement with either the Declaration of Helsinki (Tokyo, Venice, Hong Kong, Somerset West and Edinburgh amendments) or the laws and regulations of the country, whichever provides the greatest protection of the patient.

The protocol has been written, and the study will be conducted according to the ICH Harmonized Tripartite Guideline for Good Clinical Practice (ref: http://www.ifpma.org/pdfifpma/e6.pdf).

The protocol will be approved by the Local, Regional or National Ethics Committees.

## Informed consent

All patients will be informed of the aims of the study, the possible adverse events, the procedures and possible hazards to which he/she will be exposed, and the mechanism of treatment allocation. They will be informed as to the strict confidentiality of their patient data, but that their medical records may be reviewed for trial purposes by authorized individuals other than their treating physician.

It will be emphasized that the participation is voluntary and that the patient is allowed to refuse further participation in the protocol whenever he/she wants. This will not prejudice the patient’s subsequent care. Documented informed consent must be obtained for all patients included in the study before they are registered in the study. This must be done in accordance with the national and local regulatory requirements.

# Trial insurance

According to national regulations and laws, this clinical trial is covered by insurance in “Legemiddelansvarsforsikringen”. This incurance covers up to 50 patients during 2005-2007.

# Publication policy

The results of this study will be published. The main paper will discuss all aspects of the study and the author list will be as follows:

Oddbjørn Straume

Jurgen Geisler

Christian Bush

Jon Asle Bjørlykke

Torfinn Taxt

Per Eystein Lønning

Lars Andreas Akslen

After the publication of the main paper, papers on subanalysis of the results can be published with changes in the author list according to the weight and relevance of the work of each author in that specific subanalysis.

Appendix A: References

Baguley, B.C., Holdaway, K.M., Thomsen, L.L., Zhuang, L. & Zwi, L.J. (1991). Inhibition of growth of colon 38 adenocarcinoma by vinblastine and colchicine: evidence for a vascular mechanism. *Eur J Cancer*, **27,** 482-7.

Bayko, L., Rak, J., Man, S., Bicknell, R., Ferrara, N. & Kerbel, R.S. (1998). The dormant in vivo phenotype of early stage primary human melanoma: termination by overexpression of vascular endothelial growth factor. *Angiogenesis*, **2,** 203-17.

Bergsland, E. & Dickler, M.N. (2004). Maximizing the potential of bevacizumab in cancer treatment. *Oncologist*, **9 Suppl 1,** 36-42.

Bertolini, F., Paul, S., Mancuso, P., Monestiroli, S., Gobbi, A., Shaked, Y. & Kerbel, R.S. (2003). Maximum tolerable dose and low-dose metronomic chemotherapy have opposite effects on the mobilization and viability of circulating endothelial progenitor cells. *Cancer Res*, **63,** 4342-6.

Blackwell, K., Hurwitz, H., Lieberman, G., Novotny, W., Snyder, S., Dewhirst, M. & Greenberg, C. (2004). Circulating D-dimer levels are better predictors of overall survival and disease progression than carcinoembryonic antigen levels in patients with metastatic colorectal carcinoma. *Cancer*, **101,** 77-82.

Borgstrom, P., Gold, D.P., Hillan, K.J. & Ferrara, N. (1999). Importance of VEGF for breast cancer angiogenesis in vivo: implications from intravital microscopy of combination treatments with an anti-VEGF neutralizing monoclonal antibody and doxorubicin. *Anticancer Res*, **19,** 4203-14.

Burstein, H.J., Parker, L.M., Savoie, J., Younger, J., Kuter, I. & Winer, E.P. (2002). Phase II trial of the anti-VEGF antibody bevacizumab in combination with vinorelbine for refractory advanced breast cancer. *Breast Cancer Res Treat*, **76,** 115.

Chen, H.X. (2004). Expanding the clinical development of bevacizumab. *Oncologist*, **9 Suppl 1,** 27-35.

Cobleigh, M.A., Langmuir, V.K., Sledge, G.W., Miller, K.D., Haney, L., Novotny, W.F., Reimann, J.D. & Vassel, A. (2003). A phase I/II dose-escalation trial of bevacizumab in previously treated metastatic breast cancer. *Semin Oncol*, **30,** 117-24.

Dadras, S.S., Paul, T., Bertoncini, J., Brown, L.F., Muzikansky, A., Jackson, D.G., Ellwanger, U., Garbe, C., Mihm, M.C. & Detmar, M. (2003). Tumor lymphangiogenesis: a novel prognostic indicator for cutaneous melanoma metastasis and survival. *Am J Pathol*, **162,** 1951-60.

de Jong, J.S., van Diest, P.J., van der Valk, P. & Baak, J.P. (1998). Expression of growth factors, growth inhibiting factors, and their receptors in invasive breast cancer. I: An inventory in search of autocrine and paracrine loops. *J Pathol*, **184,** 44-52.

Duffaud, F. & Therasse, P. (2000). New guidelines to evaluate the response to treatment in solid tumors. *Bull Cancer*, **87,** 881-6.

Dunk, C. & Ahmed, A. (2001). Vascular Endothelial Growth Factor Receptor-2-Mediated Mitogenesis Is Negatively Regulated by Vascular Endothelial Growth Factor Receptor-1 in Tumor Epithelial Cells. *Am J Pathol*, **158,** 265-273.

Eikesdal, H.P., Bjerkvig, R. & Dahl, O. (2001). Vinblastine and hyperthermia target the neovasculature in BT(4)AN rat gliomas: therapeutic implications of the vascular phenotype. *Int J Radiat Oncol Biol Phys*, **51,** 535-44.

Erhard, H., Rietveld, F.J., van Altena, M.C., Brocker, E.B., Ruiter, D.J. & de Waal, R.M. (1997). Transition of horizontal to vertical growth phase melanoma is accompanied by induction of vascular endothelial growth factor expression and angiogenesis. *Melanoma Res*, **7 Suppl 2,** S19-26.

Fernando, N.H. & Hurwitz, H.I. (2004). Targeted therapy of colorectal cancer: clinical experience with bevacizumab. *Oncologist*, **9 Suppl 1,** 11-8.

Ferrara, N. & Davis-Smyth, T. (1997). The biology of vascular endothelial growth factor. *Endocr Rev*, **18,** 4-25.

Folkman, J., Browder, T. & Palmblad, J. (2001). Angiogenesis research: guidelines for translation to clinical application. *Thromb Haemost*, **86,** 23-33.

Gordon, M.S., Margolin, K., Talpaz, M., Sledge, G.W., Jr., Holmgren, E., Benjamin, R., Stalter, S., Shak, S. & Adelman, D. (2001). Phase I safety and pharmacokinetic study of recombinant human anti-vascular endothelial growth factor in patients with advanced cancer. *J Clin Oncol*, **19,** 843-50.

Hahnfeldt, P., Folkman, J. & Hlatky, L. (2003). Minimizing long-term tumor burden: the logic for metronomic chemotherapeutic dosing and its antiangiogenic basis. *J Theor Biol*, **220,** 545-54.

Herold-Mende, C., Steiner, H.H., Andl, T., Riede, D., Buttler, A., Reisser, C., Fusenig, N.E. & Mueller, M.M. (1999). Expression and functional significance of vascular endothelial growth factor receptors in human tumor cells. *Lab Invest*, **79,** 1573-82.

Hlatky, L., Hahnfeldt, P. & Folkman, J. (2002). Clinical application of antiangiogenic therapy: microvessel density, what it does and doesn't tell us. *J Natl Cancer Inst*, **94,** 883-93.

Hurwitz, H., Fehrenbacher, L., Novotny, W., Cartwright, T., Hainsworth, J., Heim, W., Berlin, J., Baron, A., Griffing, S., Holmgren, E., Ferrara, N., Fyfe, G., Rogers, B., Ross, R. & Kabbinavar, F. (2004). Bevacizumab plus irinotecan, fluorouracil, and leucovorin for metastatic colorectal cancer. *N Engl J Med*, **350,** 2335-42.

Johnson, D.H., Fehrenbacher, L., Novotny, W.F., Herbst, R.S., Nemunaitis, J.J., Jablons, D.M., Langer, C.J., DeVore, R.F., 3rd, Gaudreault, J., Damico, L.A., Holmgren, E. & Kabbinavar, F. (2004). Randomized phase II trial comparing bevacizumab plus carboplatin and paclitaxel with carboplatin and paclitaxel alone in previously untreated locally advanced or metastatic non-small-cell lung cancer. *J Clin Oncol*, **22,** 2184-91.

Kabbinavar, F., Hurwitz, H.I., Fehrenbacher, L., Meropol, N.J., Novotny, W.F., Lieberman, G., Griffing, S. & Bergsland, E. (2003). Phase II, randomized trial comparing bevacizumab plus fluorouracil (FU)/leucovorin (LV) with FU/LV alone in patients with metastatic colorectal cancer. *J Clin Oncol*, **21,** 60-5.

Lawson, D.H. (2004). Update on the systemic treatment of malignant melanoma. *Semin Oncol*, **31,** 33-7.

Lens, M.B. & Eisen, T.G. (2003). Systemic chemotherapy in the treatment of malignant melanoma. *Expert Opin Pharmacother*, **4,** 2205-11.

Lev, D.C., Onn, A., Melinkova, V.O., Miller, C., Stone, V., Ruiz, M., McGary, E.C., Ananthaswamy, H.N., Price, J.E. & Bar-Eli, M. (2004). Exposure of melanoma cells to dacarbazine results in enhanced tumor growth and metastasis in vivo. *J Clin Oncol*, **22,** 2092-100.

Lev, D.C., Ruiz, M., Mills, L., McGary, E.C., Price, J.E. & Bar-Eli, M. (2003). Dacarbazine causes transcriptional up-regulation of interleukin 8 and vascular endothelial growth factor in melanoma cells: a possible escape mechanism from chemotherapy. *Mol Cancer Ther*, **2,** 753-63.

Liu, B., Earl, H.M., Baban, D., Shoaibi, M., Fabra, A., Kerr, D.J. & Seymour, L.W. (1995). Melanoma cell lines express VEGF receptor KDR and respond to exogenously added VEGF. *Biochem Biophys Res Commun*, **217,** 721-7.

Lynch, T.J., Bell, D.W., Sordella, R., Gurubhagavatula, S., Okimoto, R.A., Brannigan, B.W., Harris, P.L., Haserlat, S.M., Supko, J.G., Haluska, F.G., Louis, D.N., Christiani, D.C., Settleman, J. & Haber, D.A. (2004). Activating mutations in the epidermal growth factor receptor underlying responsiveness of non-small-cell lung cancer to gefitinib. *N Engl J Med*, **350,** 2129-39.

Marcoval, J., Moreno, A., Graells, J., Vidal, A., Escriba, J.M., Garcia Ramirez, M. & Fabra, A. (1997). Angiogenesis and malignant melanoma. Angiogenesis is related to the development of vertical (tumorigenic) growth phase. *J Cutan Pathol*, **24,** 212-8.

Margolin, K., Gordon, M.S., Holmgren, E., Gaudreault, J., Novotny, W., Fyfe, G., Adelman, D., Stalter, S. & Breed, J. (2001). Phase Ib trial of intravenous recombinant humanized monoclonal antibody to vascular endothelial growth factor in combination with chemotherapy in patients with advanced cancer: pharmacologic and long-term safety data. *J Clin Oncol*, **19,** 851-6.

Mayer, R.J. (2004). Two steps forward in the treatment of colorectal cancer. *N Engl J Med*, **350,** 2406-8.

Morgan, B., Thomas, A.L., Drevs, J., Hennig, J., Buchert, M., Jivan, A., Horsfield, M.A., Mross, K., Ball, H.A., Lee, L., Mietlowski, W., Fuxuis, S., Unger, C., O'Byrne, K., Henry, A., Cherryman, G.R., Laurent, D., Dugan, M., Marme, D. & Steward, W.P. (2003). Dynamic contrast-enhanced magnetic resonance imaging as a biomarker for the pharmacological response of PTK787/ZK 222584, an inhibitor of the vascular endothelial growth factor receptor tyrosine kinases, in patients with advanced colorectal cancer and liver metastases: results from two phase I studies. *J Clin Oncol*, **21,** 3955-64.

Presta, L.G., Chen, H., O'Connor, S.J., Chisholm, V., Meng, Y.G., Krummen, L., Winkler, M. & Ferrara, N. (1997). Humanization of an anti-vascular endothelial growth factor monoclonal antibody for the therapy of solid tumors and other disorders. *Cancer Res*, **57,** 4593-9.

Ranson, M. (2004). Epidermal growth factor receptor tyrosine kinase inhibitors. *Br J Cancer*, **90,** 2250-5.

Rofstad, E.K. & Halsor, E.F. (2000). Vascular endothelial growth factor, interleukin 8, platelet-derived endothelial cell growth factor, and basic fibroblast growth factor promote angiogenesis and metastasis in human melanoma xenografts. *Cancer Res*, **60,** 4932-8.

Ruegg, C., Meuwly, J.Y., Driscoll, R., Werffeli, P., Zaman, K. & Stupp, R. (2003). The quest for surrogate markers of angiogenesis: a paradigm for translational research in tumor angiogenesis and anti-angiogenesis trials. *Curr Mol Med*, **3,** 673-91.

Rugo, H.S. (2004). Bevacizumab in the treatment of breast cancer: rationale and current data. *Oncologist*, **9 Suppl 1,** 43-9.

Salven, P., Heikkila, P. & Joensuu, H. (1997). Enhanced expression of vascular endothelial growth factor in metastatic melanoma. In *Br J Cancer*, Vol. 76. pp. 930-4.

Shields, J.D., Borsetti, M., Rigby, H., Harper, S.J., Mortimer, P.S., Levick, J.R., Orlando, A. & Bates, D.O. (2004). Lymphatic density and metastatic spread in human malignant melanoma. *Br J Cancer*, **90,** 693-700.

Simon, R.M. (1993). Design and conduct of clinical trials. In *Cancer- Principles & Parctice of Oncology*, DeVita, V.T. (ed) pp. 426. Lippincott: Philadelphia.

Sorbye, H., Glimelius, B., Berglund, A., Fokstuen, T., Tveit, K.M., Braendengen, M., Ogreid, D. & Dahl, O. (2004). Multicenter phase II study of Nordic fluorouracil and folinic acid bolus schedule combined with oxaliplatin as first-line treatment of metastatic colorectal cancer. *J Clin Oncol*, **22,** 31-8.

Srivastava, A., Laidler, P., Davies, R.P., Horgan, K. & Hughes, L.E. (1988). The prognostic significance of tumor vascularity in intermediate-thickness (0.76-4.0 mm thick) skin melanoma. A quantitative histologic study. *Am J Pathol*, **133,** 419-23.

Straume, O. & Akslen, L.A. (2001). Expression of vascular endothelial growth factor, its receptors (flt-1, kdr) and tsp-1 related to microvessel density and patient outcome in vertical growth phase melanomas. *Am J Pathol*, **159,** 223-35.

Straume, O. & Akslen, L.A. (2002). Importance of Vascular Phenotype by Basic Fibroblast Growth Factor, and Influence of the Angiogenic Factors Basic Fibroblast Growth Factor/Fibroblast Growth Factor Receptor-1 and Ephrin-A1/EphA2 on Melanoma Progression. *Am J Pathol*, **160,** 1009-1019.

Straume, O., Chappuis, P.O., Salvesen, H.B., Halvorsen, O.J., Haukaas, S.A., Goffin, J.R., Begin, L.R., Foulkes, W.D. & Akslen, L.A. (2002). Prognostic importance of glomeruloid microvascular proliferation indicates an aggressive angiogenic phenotype in human cancers. *Cancer Res*, **62,** 6808-11.

Straume, O., Jackson, D.G. & Akslen, L.A. (2003). Independent prognostic impact of lymphatic vessel density and presence of low-grade lymphangiogenesis in cutaneous melanoma. *Clin Cancer Res*, **9,** 250-6.

Straume, O., Sviland, L. & Akslen, L.A. (2000). Loss of nuclear p16 protein expression correlates with increased tumor cell proliferation (Ki-67) and poor prognosis in patients with vertical growth phase melanoma. *Clin Cancer Res*, **6,** 1845-53.

Streit, M., Velasco, P., Brown, L.F., Skobe, M., Richard, L., Riccardi, L., Lawler, J. & Detmar, M. (1999). Overexpression of thrombospondin-1 decreases angiogenesis and inhibits the growth of human cutaneous squamous cell carcinomas. *Am J Pathol*, **155,** 441-52.

Thorpe, P.E. (2004). Vascular targeting agents as cancer therapeutics. *Clin Cancer Res*, **10,** 415-27.

Valencak, J., Heere-Ress, E., Kopp, T., Schoppmann, S.F., Kittler, H. & Pehamberger, H. (2004). Selective immunohistochemical staining shows significant prognostic influence of lymphatic and blood vessels in patients with malignant melanoma. *Eur J Cancer*, **40,** 358-64.

Weidner, N. (1998). Tumoural vascularity as a prognostic factor in cancer: The evidence continues to grow. *Journal of pathology*, **184,** 119-122.

Weidner, N., Semple, J.P., Welch, W.R. & Folkman, J. (1991). Tumor angiogenesis and metastasis- correlation in invasive breast carcinoma. *N Engl J Med*, **324,** 1-8.

Willett, C.G., Boucher, Y., di Tomaso, E., Duda, D.G., Munn, L.L., Tong, R.T., Chung, D.C., Sahani, D.V., Kalva, S.P., Kozin, S.V., Mino, M., Cohen, K.S., Scadden, D.T., Hartford, A.C., Fischman, A.J., Clark, J.W., Ryan, D.P., Zhu, A.X., Blaszkowsky, L.S., Chen, H.X., Shellito, P.C., Lauwers, G.Y. & Jain, R.K. (2004). Direct evidence that the VEGF-specific antibody bevacizumab has antivascular effects in human rectal cancer. *Nat Med*, **10,** 145-7.

Yang, J.C., Haworth, L., Sherry, R.M., Hwu, P., Schwartzentruber, D.J., Topalian, S.L., Steinberg, S.M., Chen, H.X. & Rosenberg, S.A. (2003). A randomized trial of bevacizumab, an anti-vascular endothelial growth factor antibody, for metastatic renal cancer. *N Engl J Med*, **349,** 427-34.

**Appendix B:**

Patient information sheets

STUDIE 94070 (Level A)

PASIENT-INFORMASJON DEL I

Versjon 2, 09.02.05

FORESPØRSEL OM DELTAGELSE I EN STUDIE AV EFFEKT AV BEVACIZUMAB (AVASTIN) VED MALIGNT MELANOM MED SPREDNING

Les nøye gjennom pasientinformasjonen før du tar stilling, du trenger ikke svare i dag. Ta gjerne skrivet med deg hjem og rådfør deg gjerne med andre.

INTRODUKSJON

Du er blitt informert om at du har fått tilbakefall av din kreftsykdom som ikke kan opereres bort, og at din lege har anbefalt at du får medikamentell behandling for dette. Du har fått standard behandling med cellegiften dacarbazine (DTIC), men har desverre ikke hatt ønsket effekt av dette. I denne forbindelse tillater vi oss å spørre om du kunne tenke deg å være med i en studie som driver i vår avdeling, der vi undersøker behandlingseffekten av bevacizumab hos pasienter med spredning fra malignt melanom.

STANDARD BEHANDLING

Standard behandling for pasienter med spredning fra malignt melanom i dag er cellegift av typen Dacarbazine (DTIC). Denne behandlingen gir respons hos ca hver 5 pasient, men ingen pasienter kureres med denne behandlingen. Man har forsøkt å kombinere DTIC med andre typer cellegift samt med immunstimulerende medikamenter som interferon og interleukin, men studier har så langt ikke kunnet vise at dette gir bedre resultater enn DTIC alene.

ANTI-ANGIOGENESE BEHANDLING

Nyere forskning har vist hvor viktig nydannelse av blodkar (angiogenese) er for at en kreftsvulst skal vokse. Bevacizumab er et antistoff som hemmer en viktig vekstfaktor (VEGF) for nydannelse av blodkar i kreftsvulster. Bevacizumab har vist å ha behandlingseffekt ved flere typer kreftformer som tykktarmskreft og nyrekreft, men dette er ikke undersøkt for melanom.

STUDIENS MÅL

Vi vil måle behandlingseffekt av bevacizumab ved metastatisk malignt melanom. I tillegg vil vi søke å identifisere spesifikke markører hos pasienter som kan forutsi hvorvidt behandlingen virker eller ikke. Disse markørene vil bli undersøkt i vev fra svulsten, blodprøver og ved spesielle røntgenundersøkelser.

NYTTE FOR DEG?

Du vil bli tilbudt en ny type behandling for pasienter med din type sykdom, hvor vi på forhånd ikke kan si med sikkerhet om du vil ha behandlingseffekt. Så lenge du har nytte av behandlingen (stabil sykdom eller tilbakegang av sykdom) vil du kunne fortsette behandlingen. Ved tegn til vekst av sykdom stoppes behandlingen, og du vil bli tilbudt standard behandling. Undersøkelsene av markører gjøres i laboratoriet og vil ta lang tid å analysere og tolke. Dermed får disse analysene ikke direkte konsekvenser for din behandling, dog forhåpentligvis for andre pasienter i din situasjon senere.

PRAKTISK GJENNOMFØRING. BEHANDLING

Behandlingen gies som en intravenøs infusjon hver 14 dag på sengepost. Du blir undersøkt av lege hver gang du skal ha kur. Før oppstart taes en vevsprøve til forskning. Før oppstart og hver 2ndre måned taes spesialundersøkelser på røntgenavdelingen (MR, evt ultralyd og CT), og blodprøver til forskning. Det er planlagt 12 behandlinger (24 uker), pasienter som har behandlingseffekt blir tilbudt å fortsette behandlingen så lenge de har behandlingseffekt. Pasienter blir også fulgt opp med kontroller i minst 3 år etter avslutning av behandling.

Dersom du ikke ønsker å delta i studien blir du tilbudt standard behandling. Du kan også trekke deg fra studien underveis, og vil da bli tilbudt standard behandling dersom det er indikasjon for dette. Dersom du trekker deg fra studien underveis vil allerede innsamlede opplysninger inngå som studiedata, men ingen ytterligere data samles inn. Studiedata lagres i minst 15 år.

PRAKTISK GJENNOMFØRING. VEVSPRØVER TIL FORSKNING

Dersom du har en svulst som ligger like under huden, fjerner vi en del av den i lokalbedøvelse. Ved spredning til indre organer som for eksempel lever, vil man ta ut vev i en nål etter å ha satt lokalbedøvelse og generell smertestillende. Dette vil medføre ekstra stikk i tillegg til den prøven som taes ut til diagnostikk for å sikre at diagnosen er rett.

Prøvetaking av lever innebærer en liten risiko for blødning eller andre komplikasjoner, men dette er svært sjeldent og i de mer enn ti år slik teknikk har vært anvendt ved vår avdeling har vi ikke opplevd alvorlige komplikasjoner. Vevet oppbevares i biobank etter gjeldende retningslinjer frem til prosjekt slutt (01.05.2012). Vevet skal undersøkes for biologiske markører som kan si noe om behandlingen virker eller ikke.

RISIKO, BIVIRKNINGER OG UBEHAG

Klinisk bruk av bevacizumab har vist at dette er godt tolerert og gir lite plager. Likevel er det rapportert om sjeldne alvorlige bivirkninger som blødning eller sprukket tarm. Pasienter med risiko for slike komplikasjoner blir ikke tatt opp i studien. Du vil alltid kunne kontakte vår avdeling til alle døgnets tider for å få vurdert behov for innleggelse. Noe ubehag må også påregnes i forbindelse med blodprøvetaking og vevsprøvertaking.

- Kvinner i fruktbar alder må benytte sikker prevensjon. Menn må bruke sikker prevensjon. Gravide kvinner kan ikke delta i studien.

- På grunn av fare for blødning kan pasienter som bruker warfarin (Marevan®) ikke delta i studien. Det samme gjelder for pasienter som bruker daglig blodplate-hemmende betennelsesdempende medikamenter. Pasienter kan bruke acetylsalisylsyre < 325 mg x 1. Pasienter kan også bruke betennelsesdempende medikamenter mot feber og lette smerter i korte perioder og i lav dose.

- Øvrig fast medikasjon kontinueres. Du må opplyse om alle medikamenter du bruker.

DIVERSE

Deltagelse er frivillig og ditt samtykke kan trekkes tilbake uten at du trenger å oppgi grunn, og ditt forhold til behandleren påvirkes ikke av din avgjørelse.

Representanter for norske og utenlandske kontrollmyndigheter vil ha innsyn i identifiserbare data.

Kontaktperson og prosjektleder er:

Oddbjørn Straume, lege, Avdeling for Kreftbehandling og Medisinsk Fysikk, 5021 Haukeland Universitetssykehus. Tlf. 55973322, 55973866 (kveld/natt)

Behandlingsansvarlig overlege er:

Jurgen Geisler. Avdeling for Kreftbehandling og Medisinsk Fysikk, 5021 Haukeland Universitetssykehus. Tlf. 55973322

Prosjektleder har ingen økonomiske interesser eller andre interessekonflikter.

Studien er initiert av forskningsmiljøer ved Haukeland Universitetssykehus.

Pasientene er forsikret i Legemiddelansvarsforeningen.

Opplysninger om ditt sykdomsforløp blir innhentet fra pasientjournalen samt Kreftregisteret og Dødsårsaksregisteret.

Alle prosjektansatte har taushetsplikt og opplysninger behandles konfidensielt

Prosjektet har konsesjon fra Datatilsynet, og datamaterialet oppbevares i 15 år og anonymiseres innen Mai 2020

Vi ønsker å registrere opplysninger om forekomst av føflekkreft i familien. Vi forutsetter at du informerer dine pårørende og formidler et separat informasjonsskriv til de som er aktuelle for registrering.

Jeg har mottatt skriftlig og muntlig informasjon og er villig til å delta i studien

Dato: Sted: Navn: Underskrift: .

Muntlig og skriftlig informasjon er gitt av undertegnede

Dato: Sted: Navn: Underskrift: .

STUDIE 94070 (Level B)

PASIENT-INFORMASJON DEL I

Versjon 2, 09.02.05

FORESPØRSEL OM DELTAGELSE I EN STUDIE AV EFFEKT AV BEVACIZUMAB (AVASTIN) VED MALIGNT MELANOM MED SPREDNING

Les nøye gjennom pasientinformasjonen før du tar stilling, du trenger ikke svare i dag. Ta skrivet med deg hjem og rådfør deg gjerne med andre.

INTRODUKSJON

Du er blitt informert om at du har fått tilbakefall av din kreftsykdom som ikke kan opereres bort, og at din lege anbefaler at du får medikamentell behandling for dette. I denne forbindelse tillater vi oss å spørre om du kunne tenke deg å være med i en studie som driver i vår avdeling, der vi undersøker behandlingseffekten av bevacizumab hos pasienter med spredning fra malignt melanom.

STANDARD BEHANDLING

Standard behandling for pasienter med spredning fra malignt melanom i dag er cellegift av typen Dacarbazine (DTIC). Denne behandlingen gir respons hos ca hver 5 pasient, men ingen pasienter kureres av denne behandlingen. Man har forsøkt å kombinere DTIC med andre typer cellegift samt med immunstimulerende medikamenter som interferon og interleukin, men studier har så langt ikke kunnet vise at dette gir bedre resultater enn DTIC alene.

ANTI-ANGIOGENESE BEHANDLING

Nyere forskning har vist hvor viktig nydannelse av blodkar (angiogenese) er for at en kreftsvulst skal vokse. Bevacizumab er et antistoff som hemmer en viktig vekstfaktor (VEGF) for nydannelse av blodkar i kreftsvulster. Bevacizumab har vist å ha behandlingseffekt ved flere typer kreftformer som tykktarmskreft og nyrekreft, men dette er ikke undersøkt for melanom.

STUDIENS MÅL

Vi vil måle behandlingseffekt av bevacizumab ved metastatisk malignt melanom. I tillegg vil vi søke å identifisere spesifikke markører hos pasienter som kan forutsi hvorvidt behandlingen virker eller ikke. Disse markørene vil bli undersøkt i vev fra svulsten, blodprøver og ved spesielle røntgenundersøkelser.

NYTTE FOR DEG?

Du vil bli tilbudt en ny type behandling for pasienter med din type sykdom, hvor vi på forhånd ikke kan si med sikkerhet om du vil ha behandlingseffekt. Så lenge du har nytte av behandlingen (stabil sykdom eller tilbakegang av sykdom) vil du kunne fortsette behandlingen. Ved tegn til vekst av sykdom, vil du straks bli tilbudt standard behandling med DTIC dersom du ikke har fått det før. Undersøkelsene av markører gjøres i laboratoriet og vil ta lang tid å analysere og tolke. Dermed får disse analysene ikke direkte konsekvenser for din behandling, dog forhåpentligvis for andre pasienter i din situasjon senere.

PRAKTISK GJENNOMFØRING. BEHANDLING

Behandlingen gies som en intravenøs infusjon hver 14 dag på sengepost. Du blir undersøkt av lege hver gang du skal ha kur. Før oppstart taes en vevsprøve til forskning. Før oppstart og hver 2ndre måned taes spesialundersøkelser på røntgenavdelingen (MR, evt ultralyd og CT), og blodprøver til forskning. Det er planlagt 12 behandlinger (24 uker), pasienter som har behandlingseffekt blir tilbudt å fortsette behandlingen så lenge de har behandlingseffekt. Pasienter blir også fulgt opp med kontroller i minst 3 år etter avslutning av behandling.

Dersom du ikke ønsker å delta i studien blir du tilbudt standard behandling. Du kan også trekke deg fra studien underveis, og vil da bli tilbudt standard behandling dersom det er indikasjon for dette. Dersom du trekker deg fra studien underveis vil allerede innsamlede opplysninger inngå som studiedata, men ingen ytterligere data samles inn. Studiedata lagres i minst 15 år.

PRAKTISK GJENNOMFØRING. VEVSPRØVER TIL FORSKNING

Dersom du har en svulst som ligger like under huden, fjerner vi en del av den i lokalbedøvelse. Ved spredning til indre organer som for eksempel lever, vil man ta ut vev i en nål etter å ha satt lokalbedøvelse og generell smertestillende. Dette vil medføre ekstra stikk i tillegg til den prøven som taes ut til diagnostikk for å sikre at diagnosen er rett.

Prøvetaking av lever innebærer en liten risiko for blødning eller andre komplikasjoner, men dette er svært sjeldent og i de mer enn ti år slik teknikk har vært anvendt ved vår avdeling har vi ikke opplevd alvorlige komplikasjoner. Vevet oppbevares i biobank etter gjeldende retningslinjer frem til prosjekt slutt (01.05.2012). Vevet skal undersøkes for biologiske markører som kan si noe om behandlingen virker eller ikke.

RISIKO, BIVIRKNINGER OG UBEHAG

Klinisk bruk av bevacizumab har vist at dette er godt tolerert og gir lite plager. Likevel er det rapportert om sjeldne alvorlige bivirkninger som blødning eller sprukket tarm. Pasienter med risiko for slike komplikasjoner blir ikke tatt opp i studien. Du vil alltid kunne kontakte vår avdeling til alle døgnets tider for å få vurdert behov for innleggelse. Noe ubehag må også påregnes i forbindelse med blodprøvetaking og vevsprøvertaking.

- Kvinner i fruktbar alder må benytte sikker prevensjon. Menn må bruke sikker prevensjon. Gravide kvinner kan ikke delta i studien.

- På grunn av fare for blødning kan pasienter som bruker warfarin (Marevan®) ikke delta i studien. Det samme gjelder for pasienter som bruker daglig blodplate-hemmende betennelsesdempende medikamenter. Pasienter kan bruke acetylsalisylsyre < 325 mg x 1. Pasienter kan også bruke betennelsesdempende medikamenter mot feber og lette smerter i korte perioder og i lav dose.

- Øvrig fast medikasjon kontinueres. Du må opplyse om alle medikamenter du bruker.

DIVERSE

Deltagelse er frivillig og ditt samtykke kan trekkes tilbake uten at du trenger å oppgi grunn, og ditt forhold til behandleren påvirkes ikke av din avgjørelse.

Representanter for norske og utenlandske kontrollmyndigheter vil ha innsyn i identifiserbare data.

Kontaktperson og prosjektleder er:

Oddbjørn Straume, lege, Avdeling for Kreftbehandling og Medisinsk Fysikk, 5021 Haukeland Universitetssykehus. Tlf. 55973322, 55973866 (kveld/natt)

Behandlingsansvarlig overlege er:

Jurgen Geisler. Avdeling for Kreftbehandling og Medisinsk Fysikk, 5021 Haukeland Universitetssykehus. Tlf. 55973322

Prosjektleder har ingen økonomiske interesser eller andre interessekonflikter.

Studien er initiert av forskningsmiljøer ved Haukeland Universitetssykehus.

Pasientene er forsikret i Legemiddelansvarsforeningen.

Opplysninger om ditt sykdomsforløp blir innhentet fra pasientjournalen samt Kreftregisteret og Dødsårsaksregisteret.

Alle prosjektansatte har taushetsplikt og opplysninger behandles konfidensielt

Prosjektet har konsesjon fra Datatilsynet, og datamaterialet oppbevares i 15 år og anonymiseres innen Mai 2020

Vi ønsker å registrere opplysninger om forekomst av føflekkreft i familien. Vi forutsetter at du informerer dine pårørende og formidler et separat informasjonsskriv til de som er aktuelle for registrering.

Jeg har mottatt skriftlig og muntlig informasjon og er villig til å delta i studien

Dato: Sted: Navn: Underskrift: .

Muntlig og skriftlig informasjon er gitt av undertegnede

Dato: Sted: Navn: Underskrift: .

STUDIE 94070

PASIENT-INFORMASJON DEL II-a

Versjon 3, 04.04.05

Pasientens eksemplar

GENTESTING

I forbindelse med gentesting snakker vi i prinsippet om to forskjellige testformer. Det ene er å undersøke på forandringer i selve svulstvevet som i utgangspunktet ikke har noe med nedarvede genforandringer og disposisjon for sykdom å gjøre, men er en del av utviklingen av den spesifikke kreftsvulsten. På den andre siden snakker vi om nedarvede genforandringer som kan disponere for kreftsykdom. Slike gener har man i dag identifisert i relasjon til enkelte sykdommer som blant annet brystkreft. Hva angår malignt melanom har man imidlertid ikke kunnet identifisere sikre genforandringer som disponerer for denne sykdommen.

Hovedhensikten med dette prosjektet er å se på forandringer i selve svulstvevet i relasjon til terapieffekt og ikke se på mulige nedarvede mutasjoner. Med den rivende utvikling som er innenfor feltene genetikk og kreft ser vi imidlertid for oss at under gjennomføringen av dette prosjektet kan det muligens komme data fra andre sentre som viser at spesielle genforandringer i normalvevet øker risikoen for malignt melanom. Hvis samtidig slike genforandringer antas å være av betydning for behandlingseffekten av svulstene, kan det være aktuelt å se på dem i svulsten og, dersom vi finner genforandringen der, også se om den finnes i normalvevet hos den enkelte pasient.

Hensikten med å se på forandringen også i det normale arvestoffet vil i så tilfelle være at slik informasjon kan være av betydning for å utvikle og forbedre behandlingen av sykdommen. Selv om slike genforandringer ikke er kjent i dag, ser vi for oss at vi kan komme i en situasjon med dette prosjektet der det kan være ønskelig å se på slike mulige endringer i prøver tatt fra enkelte pasienter.

Informasjon fra en slik gentest vil potensielt ikke bare kunne ha betydning for den enkelte pasient men vil også kunne ha betydning for å vurdere risiko for sykdomsutvikling hos andre familiemedlemmer. Det er derfor forbundet med etiske problemer å foreta slik testing.

I og med at hensikten med studien ikke er å kartlegge eventuelt risikogener og arvelig disposisjon for malignt melanom men å se på genforandringer i forbindelse med behandlingseffekt, er det sannsynlig at vi ikke vil komme opp i slike problemstillinger i det hele tatt. Vi ønsker imidlertid, dersom det skulle bli aktuelt, å ha innhentet tillatelse på forhånd til å foreta en slik analyse. Siden resultatene av studien ikke vil virke direkte inn på din behandling vil vi i utgangspunktet ikke opplyse pasient eller pårørende om at en genanalyse er gjort og hva som ble resultatet av den. Pasient eller nærmeste pårørende vil kunne henvende seg direkte til oss få disse opplysningene dersom dette inngår som ledd i utredning fra en genetisk avdeling. Råd om dette vil en kunne få ved å kontakte Avdeling for Medisinsk Genetikk ved Haukeland Universitetssykehus. På denne måten vil vi kunne stille til rådighet informasjon dersom en familie er aktuell for genetisk utredning, samtidig som ingen vil bli påtvunget informasjon som de selv ikke aktivt søker. Vi presiserer at slik informasjon overhodet ikke vil bli stilt til rådighet for noen utenforstående, idet den ikke blir skrevet inn i journalen. Ei heller på forespørsel (forsikringsselskaper etc.). Informasjonen vil kun stilles til rådighet for pasient eller nærmeste pårørende dersom de eksplisitt ber om det.

Pasienten oppretter førstegangskontakt med relevante familiemedlemmer, og formidler en egen samtykkeerklæring til disse.

SAMTYKKEERKLÆRING

Undertegnede: …………………………………………………Født: …………….

har lest ovenstående informasjon. På bakgrunn av denne tillater jeg at de også kan analysere på mulige genforandringer i mitt normalvev (blodprøver) så lenge undersøkerene har forpliktet seg til å håndtere informasjonen på ovenstående måte

Dato: ..…… Underskrift pasient: ……………………............... Underskrift lege: …………………….............

STUDIE 94070

PASIENT-INFORMASJON DEL II-B

Versjon 1, 04.04.05

Pårørendes eksemplar

FORSKNING PÅ ARVELIG FØFLEKKREFT

Siden du er pårørende av en av våre pasienter som vi ønsker å ha med i en forskningsprotokoll hvor det inngår gentesting **av pasienten**, ber vi deg om å lese gjennom denne informasjonen, og evt. signere for samtykke til at vi kan registrere opplysninger også om din sykdom.

Hovedhensikten med studien er å se på genforandringer i forbindelse med behandlingseffekt, og *ikke* å kartlegge eventuelt risikogener og arvelig disposisjon for malignt melanom. Skulle vi likevel komme på sporet av slike genskader ønsker vi å ha innhentet tillatelse på forhånd til å foreta en registrering om din sykdom. Siden resultatene av studien ikke vil virke direkte inn på din pårørendes behandling vil vi i utgangspunktet ikke opplyse pasient eller pårørende om at en genanalyse er gjort og hva som ble resultatet av den. Pasient eller nærmeste pårørende vil kunne henvende seg direkte til oss få disse opplysningene dersom dette inngår som ledd i utredning fra en genetisk avdeling. Råd om dette vil en kunne få ved å kontakte Avdeling for Medisinsk Genetikk ved Haukeland Universitetssykehus. På denne måten vil vi kunne stille til rådighet informasjon dersom en familie er aktuell for genetisk utredning, samtidig som ingen vil bli påtvunget informasjon som de selv ikke aktivt søker. Vi presiserer at slik informasjon overhodet ikke vil bli stilt til rådighet for noen utenforstående, idet den ikke blir skrevet inn i journalen. Ei heller på forespørsel (forsikringsselskaper etc.). Informasjonen vil kun stilles til rådighet for pasient eller nærmeste pårørende dersom de eksplisitt ber om det.

DIVERSE

Registreringen er frivillig og ditt samtykke kan trekkes tilbake uten at du trenger å oppgi grunn, og pasientens forhold til behandleren påvirkes ikke av din avgjørelse.

Representanter for norske og utenlandske kontrollmyndigheter vil ha innsyn i identifiserbare data.

Kontaktperson og prosjektleder er:

Oddbjørn Straume, lege, Avdeling for Kreftbehandling og Medisinsk Fysikk, 5021 Haukeland Universitetssykehus. Tlf. 55973322, 55973866 (kveld/natt)

- Behandlingsansvarlig overlege er:

Jurgen Geisler. Avdeling for Kreftbehandling og Medisinsk Fysikk, 5021 Haukeland Universitetssykehus. Tlf. 55973322

Prosjektleder har ingen økonomiske interesser eller andre interessekonflikter.

Studien er initiert av forskningsmiljøer ved Haukeland Universitetssykehus.

Opplysninger om ditt sykdomsforløp blir formidlet av pasienten.

Alle prosjektansatte har taushetsplikt og opplysninger behandles konfidensielt

Prosjektet har konsesjon fra Datatilsynet, og datamaterialet oppbevares i 15 år og anonymiseres innen Mai 2020

Vi presiserer at vi kun ønsker å registrere opplysninger om din sykdom, vi skal IKKE gjøre genanalyser på prøver tatt fra deg.

SAMTYKKEERKLÆRING

Undertegnede: …………………………………………………Født: …………….

Familierelasjon:...........................av pasienten (navn):..............................................................................................

har lest ovenstående informasjon. På bakgrunn av denne tillater jeg at de kan innhente og registrere informasjon om min melanom sykdom så lenge undersøkerene har forpliktet seg til å håndtere informasjonen på ovenstående måte. Pasienten formidler opplysningene til lege tilknyttet prosjektet.

Dato: ..…… Underskrift pårørende: ……………………............... Underskrift lege: ………………….............
